# Cascade Mesophase Transitions of Multi-enzyme Responsive Polymeric Formulations

**DOI:** 10.1021/acs.biomac.4c00221

**Published:** 2024-05-22

**Authors:** Parul Rathee, Nicole Edelstein-Pardo, Gil Koren, Roy Beck, Roey J. Amir

**Affiliations:** † School of Chemistry, Faculty of Exact Sciences, Tel-Aviv University, Tel-Aviv 6997801, Israel; ‡ The Center for Physics and Chemistry of Living Systems, Tel-Aviv University, Tel Aviv 6997801, Israel; § Center for Nanoscience and Nanotechnology, Tel-Aviv University, Tel Aviv 6997801, Israel; ∥ School of Physics and Astronomy, Faculty of Exact Sciences, Tel-Aviv University, Tel-Aviv 6997801, Israel; ⊥ ADAMA Center for Novel Delivery Systems in Crop Protection, Tel-Aviv University, Tel Aviv 6997801, Israel

## Abstract

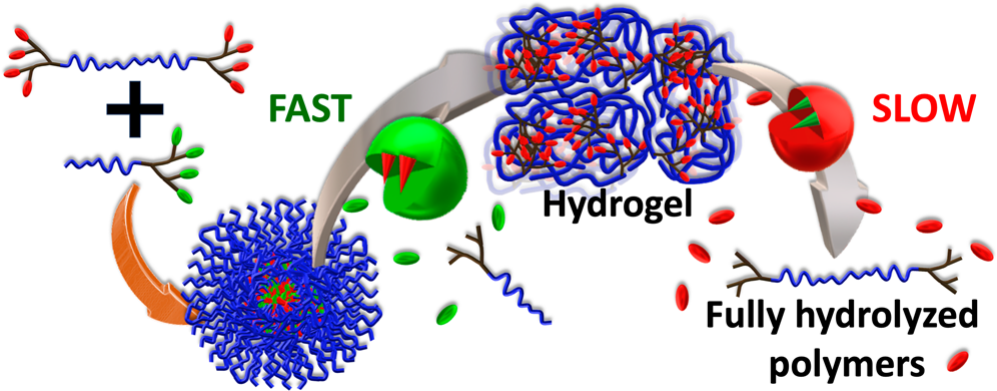

Studying how synthetic polymer assemblies respond to sequential enzymatic stimuli can uncover intricate interactions in biological systems. Using amidase- and esterase-responsive PEG-based diblock (DBA) and triblock amphiphiles (TBAs), we created two distinct formulations: amidase-responsive DBA with esterase-responsive TBA and vice versa. We studied their cascade responses to the two enzymes and the sequence of their introduction. These formulations underwent cascade mesophase transitions upon the addition of the DBA-degrading enzyme, transitioning from (i) coassembled micelles to (ii) triblock-based hydrogel, and ultimately to (iii) dissolved polymers when exposed to the TBA hydrolyzing enzyme. The specific pathway of the two mesophase transitions depended on the compositions of the formulations and the enzyme introduction sequence. The results highlight the potential for designing polymeric formulations with programmable multistep enzymatic responses, mimicking the complex behavior of biological macromolecules.

## Introduction

Many biological macromolecules, such as proteins and nucleic acids, can respond by different pathways to complex combinations of environmental factors and stimuli.^1,2^ To replicate and understand this complexity, researchers have been working on creating smart materials that can sense and respond to multiple stimuli in a predictable manner.^3−8^ Stimuli-responsive polymers, often called “smart polymers”, are fascinating materials with unique properties that allow them to adapt and respond to changes in their surrounding environment^9−12^ such as pH,^13,14^ light,^15−19^ temperature,^20−23^ redox,^24^ external magnetic field,^25^ and enzymatic action.^26−32^ Within this category, multiresponsive polymeric materials have gained substantial attention over the years for their ability to respond to two or more types of stimuli. Lately, there have been numerous reports discussing the integration of dual-stimuli-responsive functionalities into a single polymer system, making it possible to mimic the complex behavior of natural systems. Some notable examples encompass polymers that are temperature- and pH-responsive,^33−35^ pH- and reduction-responsive,^36−38^ photo- and temperature-responsive,^39,40^ enzyme- and temperature-responsive,^41^ photo- and enzyme-responsive,^42^ and others.^43−46^ Despite these advancements, there are only a few studies focused on dual-stimuli-responsive systems that can respond to two different types of enzymes.^47^ Previously, we combined two PEG-dendron diblock amphiphiles (DBAs), each with a distinct type of enzymatically cleavable hydrophobic end-groups. The coassembled micelles could undergo full enzymatically induced disassembly only in the presence of both activating enzymes.^48^ More recently, we have expanded our studies to dendron-PEG-dendron triblock amphiphiles (TBAs) and used these TBAs to form electrospun fibers, which were found to swell into hydrogels in an aqueous media.^49^ However, when mixing DBA and TBA, which had the same hydrophilic to hydrophobic ratio and identical hexanoate end-groups, these two amphiphilic architectures coassembled into nanosized micelles.^50^ Due to the faster unimermicelle exchange rate of DBA, it selectively undergoes cleavage by porcine liver esterase (PLE), serving as a model enzyme capable of cleaving the hexanoate end-groups, leaving TBA intact. This change in the ratio between DBA and TBA in the micelles leads to the transition of TBA to a hydrogel. The formed hydrogel can finally be degraded by PLE to fully hydrolyzed hydrophilic polymers. Aiming to enhance the complexity of this mixed micellar formulation and their enzymatically induced transition between three sequential mesophases: (i) micelles to (ii) hydrogel, and finally to (iii) dissolved polymers, we decided to incorporate amphiphiles with two different enzyme-responsive groups. We wished to study how these coassembled micelles would respond to the sequences in which the two activating enzymes will be introduced. We believe that this strategy holds great promise in mirroring the complex behavior of biological macromolecules in nature.

## Experimental Section

### Instrumentation

HPLC: All measurements were recorded on a Waters Alliance e2695 separation module equipped with a Waters 2998 photodiode array detector. All solvents were purchased from Bio-Lab Chemicals and were used as received. All solvents are of HPLC grade. NMR: Spectra were recorded on a Bruker AVANCE III 400 MHz/100 MHz spectrometer. Chemical shifts are reported in ppm and referenced to the solvent. The molecular weights of the dendron-PEG-dendron triblock copolymers were determined by comparison of the areas of the peaks corresponding to the PEG block (3.63 ppm) and the proton peaks of the dendrons. SEC: All measurements were recorded on Viscotek GPC max by Malvern using a refractive index detector and PEG standards (purchased from Sigma-Aldrich) were used for calibration. DMF (purchased from Sigma, HPLC grade) was used as the mobile phase. Columns (2 × PSS GRAM 1000 Å) were used at a column temperature of 50 °C. DLS: All measurements were recorded on a Corduan Technology VASCO^γ^ particle size analyzer. Absorbance Spectra: All measurements were recorded on a TECAN Infinite M200Pro device using a quartz plate. Rheometer: Rheological measurements were performed using a controlled-stress rheometer (AR-G2, TA Instruments, USA). An 8 mm diameter flat-plate geometry with a cross-hatched surface was used for the study.

### Materials

Poly(ethylene glycol) (10 kDa), poly(ethylene glycol) methyl ether (5 kDa), allyl bromide (99%), 2,2-dimethoxy-2-phenylacetophenone (99%), Fmoc-Lys(Boc)-OH Novabiochem, *N*,*N*′-diisopropylcarbodiimide, propargyl bromide, 80% solution in toluene, 4-nitrophenol (99.5%), 4-dimethylamino pyridine, *N*,*N*′-dicyclohexylcarbodiimide (99%), 2-mercaptoethanol, penicillin G amidase from *Escherichia coli* (PGA), PLE, hexanoic acid, triethylsilane, phenyl acetyl chloride, and Sephadex LH20 were purchased from Sigma-Aldrich. Anhydrous potassium carbonate and trifluoroacetic acid were purchased from Alfa Aesar. Potassium hydroxide, cystamine hydrochloride, Oxyma Pure Novabiochem Triphenylmethyl chloride, diisopropylethylamine (DIPEA), and bovine serum albumin (BSA, Probumin) were purchased from Merck. 2-(1*H*-Benzotriazole-1-yl)-1,1,3,3-tetramethyluronium hexafluorophosphate was purchased from Chem-Impex. Piperidine, Silica Gel 60 Å 0.040–0.063 mm, sodium hydroxide, and all solvents were purchased from Bio-Lab and were used as received. Deuterated solvents for NMR were purchased from Cambridge Isotope Laboratories (CIL). DBA and TBA were synthesized as detailed in the Supporting Information.

### General Procedure for Coassembled Micellar Formulation Preparation

5 mg of respective DBA and 5 mg of respective TBA was mixed together in acetonitrile to obtain a maximum blending, giving a total polymer concentration of 10 mg/mL. The sample was vortexed and sonicated until full solubility was obtained, followed by evaporation of the solvent to yield a thin film. This thin film was then hydrated with 1 mL of PBS buffer resulting in the formation of coassembled micelles.

### Transmission Electron Microscopy

Preparation of Samples: A micellar solution of DBA was prepared by mixing 5 mg of DBA (Ester-DBA or Amide-DBA) in 1 mL of PBS to give a polymer concentration of 5 mg/mL. For coassembled micellar formulations, 5 mg of respective DBA and TBA was mixed together to give a total polymer concentration of 10 mg/mL. Measurement: 30 μL of the solution was dropped onto carbon-coated copper grids. The excessive solvent of the droplet was wiped away using filter paper, and the sample grids were left to dry in air at RT. Then, grids were inspected in a transmission electron microscope (TEM), operated at 2000 kV.

### Enzymatic Degradation Experiments

Amide-DBA and amide-TBA formulation: PGA stock solution was added into the formulation solution to yield a final concentration of 0.52 U/mL. Amide-DBA and ester-TBA formulation: PGA stock solution was added into the formulation solution to yield a final concentration of 0.52 U/mL and after 6 h PLE stock solution was added into the same solution to yield a final concentration of 10 U/mL. Ester-DBA and amide-TBA formulation: PLE stock solution was added into the formulation solution to yield a final concentration of 3.5 U/mL and after 6 h PGA stock solution was added into the same solution to yield a final concentration of 2.62 U/mL. The degradation for all three formulations was followed at 37 °C by monitoring the area under the peak of the parent DBA, TBA, and hydrolyzed polymer by HPLC at 297 and 423 nm.

For absorption measurements, we diluted 10 μL of the micellar solution with 240 μL of acetonitrile, followed by centrifugation. The absorption spectra of the mixed labeled micelles were collected over a range of 300–600 nm in a 96-well quartz plate. We followed the same protocol every hour, spanning up to 10 h, for all three formulations.

### Rheology Measurements

Rheological measurements of the aggregated hydrogels were performed using a controlled-stress rheometer (AR-G2, TA Instruments, USA). An 8 mm diameter flat-plate geometry with a rough surface was used for the study. The viscous elastic region was determined by strain sweep from 0.01 to 100% strain at 1 Hz frequency at 25 °C with a gap size of 0.9 mm.

### High-Resolution Scanning Electron Microscope

We use a Zeiss Gemini 300 high-resolution scanning electron microscope (HRSEM) in high vacuum, 15 kV, to image the internal structure of the different hydrogels formed. The supernatant left after conducting the enzymatic degradation experiments was removed, and the hydrogel aggregated at the bottom of the vial was washed with PBS. The gel samples were freeze-dried in liquid nitrogen and then adhered with carbon tape onto SEM stubs. All samples were sputter coated with a thin layer of (approximately 10 nm) Au prior to imaging.

### Hydrogel Degradation

Two parallel experiments were conducted to study the stability of the aggregated hydrogels for all three different formulations. In the first experiment, 500 μL of 3.5 mg/mL BSA in PBS was added to the gel. In the second experiment, 500 μL of 3.5 mg/mL BSA, along with a higher concentration of enzyme in PBS, was added. For the amide-based gel, PGA was added to yield a final concentration of 9.17 U/mL, while, for the ester-based gel, PLE was added to achieve a final concentration of 35 U/mL.

## Results and Discussion

**Scheme 1:**
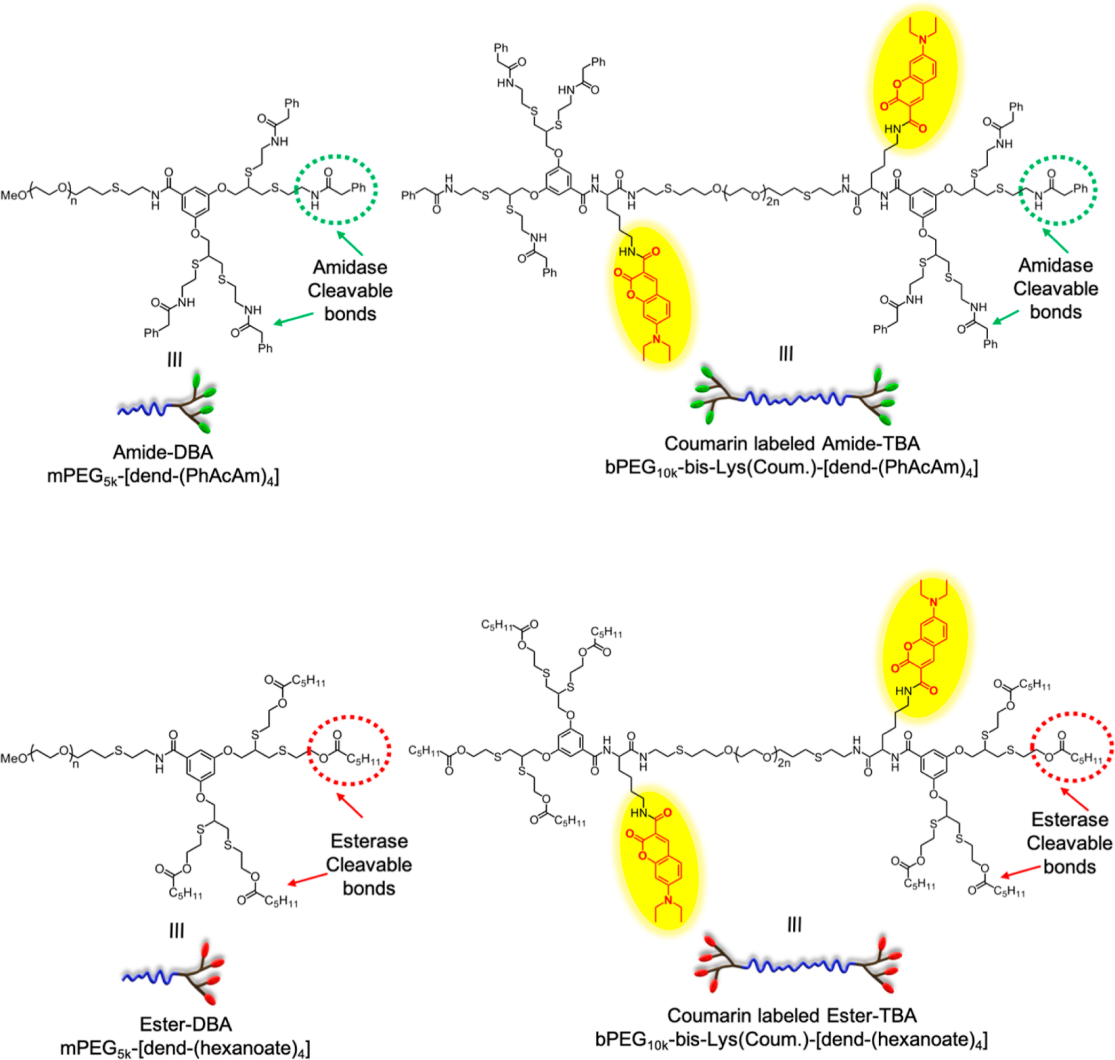
Structures of DBAs and Coumarin-Labeled TBAs

To prepare the coassembled micellar formulations, we started by synthesizing two types of DBAs and two types of TBAs. Both DBAs were based on a commercial 5 kDa monofunctional poly(ethene glycol) (mPEG) as a hydrophilic block and a dendron with four enzymatically cleavable end-groups as the hydrophobic block. The first DBA, termed amide-DBA, contained four phenylacetamide end-groups that can be cleaved by PGA. The second DBA, termed ester-DBA, was functionalized with esterase-cleavable hexanoate end-groups. The two DBAs were synthesized by a convergent approach starting from a PEG-amine, as previously reported.^51^ The amide-TBA and ester-TBA were composed of two amidase- and esterase-cleavable dendrons, respectively, as the hydrophobic side block and a 10 kDa bifunctional polyethene glycol (bPEG), precisely twice the molecular weight of the mPEG chain used for preparing the DBAs, as the middle hydrophilic block (Scheme 1).

To better visualize and track the formation of triblock-based hydrogels that were expected to form upon enzymatic activation, we labeled the two TBAs with 7-(diethylamino) coumarin-3-carboxylic acid, which has a distinctive absorbance maximum at around 420 nm.

The synthesis of TBAs followed a similar methodology but also included the insertion of a lysine-based trilinker between the bPEG and the dendrons, which was later used for labeling. The detailed synthesis of all four amphiphiles is shown in the Supporting Information (Schemes S3 and S4). The synthesized amphiphiles were characterized by NMR, HPLC, and size exclusion chromatography (SEC) to verify their synthetic conversion, purity, and polydispersity. The experimental results are in good agreement with the expected values, as can be seen in Supporting Information and Table 1.

**Table 1:** Amide and Ester Functionalized Amphiphiles and Their Properties

amphiphile	end-group	*M*_n_^a^ (kDa)	*D̵*_M_^b^	calc. *M*_n_^c^ (kDa)	cmc^d^ (μM)	*D*_H_^e^ (nm)	clog *p*^f^
amide-DBA	phenyl acetamide	6.0	1.05	6.1	21 ± 2	11 ± 2	6.3
amide-TBA	phenyl acetamide	12.8	1.15	13.0			8.3
ester-DBA	hexanoate	5.9	1.05	6.0	6 ± 1	13 ± 3	11.6
ester-TBA	hexanoate	12.9	1.08	12.8			13.6

a Measured by SEC using PEG commercial standards.

b Polydispersity index.

c Calculated based on mPEG_5 kDa_ or bPEG10_kDa_ and the expected exact mass of the dendrons.

d Determined using the Nile red method.

e Hydrodynamic diameter measured by DLS.

f Calculated for only the dendritic group of the amphiphile via ChemDraw Version 22.0.

After synthesizing and characterizing all four polymeric amphiphiles, we first checked their self-assembly behavior in aqueous media (PBS pH = 7.4). Both amide-DBA and ester-DBA formed micelles with diameters of around 12 nm, as measured by using dynamic light scattering (DLS) (Figure S14). Further validation for the formation of micelles from the two types of DBAs was obtained by transmittance electron microscopy (TEM), which showed spherical structures with similar diameters (Figures S15A,B). The critical micelle concentrations (cmcs) for the DBAs were determined using the Nile red method.^52^ The cmcs were found to be around 21 μM for amide-DBA and 6 μM for ester-DBA, attributed to the more hydrophobic nature of the esters containing dendrons (Table 1 and Figures S9 and S10). As expected, in contrast to the DBAs and although having identical hydrophilic to hydrophobic ratios, the two TBAs formed hydrogels when dissolved in PBS (Figure S31).

### Amide-DBA and Amide-TBA Formulation

As we recently demonstrated the ability of ester-based DBA and TBA formulations to undergo enzymatic induced transition from micelles to hydrogel and then to soluble polymers,^50^ we wished here to first evaluate the ability of the amide-DBA and amide-TBA formulation to coassemble into mixed micelles and examine if their enzymatic activation would result in a similar cascade of mesophase transitions. Based on our previous study of ester-based formulation, the enzymatic cleavage of the coassembled micelles should selectively lead to the degradation of amide-DBA into the corresponding hydrolyzed PEG-dendrons and the transition of amide-TBA into hydrogel, as illustrated schematically in Figure 2A.

**Figure 1:**
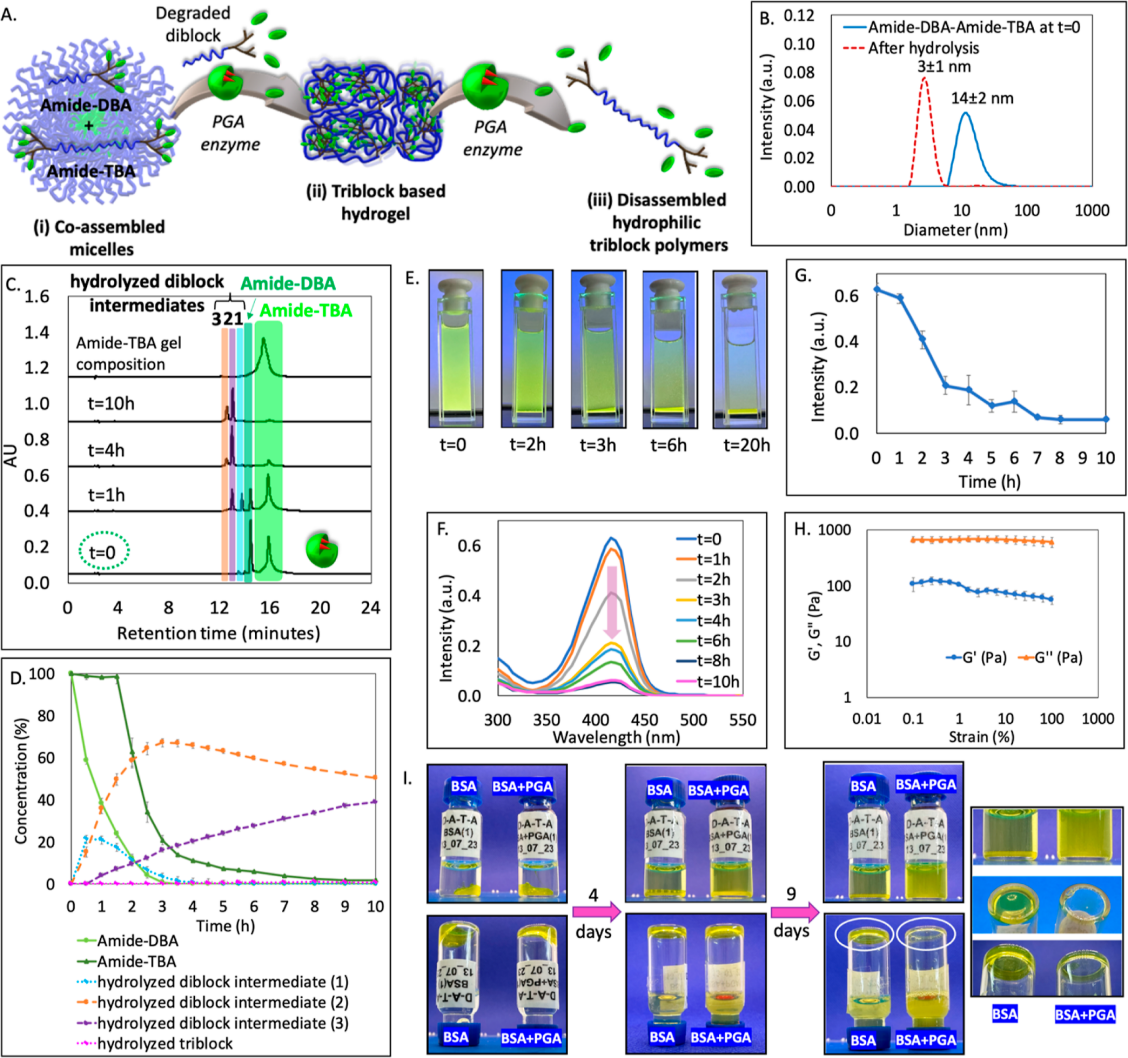
(A) Schematic illustration of the mesophase transition of amide-DBA and amide-TBA formulation from (i) coassembled mixed micelles into a (ii) hydrogel and finally to (iii) fully hydrolyzed polymers. (B) DLS of micelle solution before and after the addition of PGA. (C) Overlay of HPLC chromatograms before and after the addition of PGA, and the dissolved TBA-based hydrogel. (D) HPLC-based kinetic data. (E) Photos of the solution at different time points before and after the addition of PGA. (F) Time-dependent absorbance spectra and (G) maximum intensity at 415 nm as a function of time. (H) Amplitude sweep test of the hydrogel at a constant frequency of 1 Hz. (I) Photos of the vials containing hydrogel with BSA or BSA and PGA over 13 days, indicating the transformation of hydrogel into hydrolyzed polymers in the presence of PGA (on the right—zoomed-in photos taken after 13 days).

We started by mixing amide-DBA and amide-TBA at a 1:1 weight ratio in an organic solvent to obtain maximal blending, followed by evaporation of the solvent to yield a thin film. Hydration of this film using PBS^53,54^ resulted in the desired formation of micelles with a diameter of around 14 nm as indicated by DLS and TEM (Figure 1B, solid line, and S15C). The cmc for this formulation was found to be approximately 5 μM (Figure S11). This decrease by a factor of 4 in the cmc in comparison with the amide-DBA alone points out the greater thermodynamic stability of the coassembled micelles. After the formation of mixed micelles was confirmed, our aim was to monitor their enzymatic activation process using a combination of HPLC and absorption spectroscopy. HPLC was used to directly monitor the enzymatic degradation of the two types of amphiphiles that form the coassembled micellar solution. Additionally, absorption spectroscopy enabled us to track the transition of the micelles into the hydrogel, which precipitated, by monitoring the intensity of conjugated coumarin dye, as the amide-TBA aggregated into hydrogel. The micellar solution was incubated with the PGA enzyme and we directly monitored its molecular composition using HPLC (Figure 1C). Initially, two peaks corresponding to the two types of amphiphiles in the expected 1:1 ratio were observed. Similar to our recent report on esterase-responsive coassembled micelle, we also noted a high selectivity of the enzyme toward the degradation of amide-DBA, which reached nearly 76% after 1.5 h, while TBA remained nearly intact (with 2% degradation). Additionally, we observed that not all end-groups of amide-DBA were cleaved and the formation of partially cleaved intermediates was observed. This observation correlates well with our previous studies on enzymatic degradation of hybrids containing phenylacetamide end-groups.^44^ After 2 h, we observed a sudden drop of approximately 40% in the TBA concentration (Figure 1D). The chromatograms did not indicate an extensive simultaneous formation of the cleaved triblock, implying that the disappearance of amide-TBA is not a result of its enzymatic degradation but rather due to its aggregation into a hydrogel, as depicted in pictures taken at different time points (Figure 1E). This mesophase transition and the precipitation of the formed hydrogel particles explain the rapid decrease in the concentration of amide-TBA in the solution. After 10 h, the starting amide-DBA was completely degraded and only PEG-dendrons with two or three cleaved end-groups were observed. DLS measurement conducted thereafter for the solution did not show any presence of micelles as well (Figure 1B, dashed line). To analyze the composition of the formed hydrogels, we dissolved them in acetonitrile, which is a good solvent for both blocks, and analyzed the organic solution by HPLC (Figure 1C), which showed the expected presence of intact amide-TBA. To further monitor this mesophase transition from micelles to hydrogel, we also measured the absorbance of the samples, utilizing the coumarin marker attached to our amide-TBA. Absorption spectra of the mixed labeled micelles were collected over a range of 300–600 nm. The results showed a maximum at 415 nm (Figure 1F). Subsequently, we followed the decrease in absorbance upon adding the activating enzyme to the mixed micellar formulation. The absorbance exhibited minimal change during the first hour but considerably decreased in the next 2 h, followed by a slower decrease thereafter (Figure 1G). This observation reaffirms the gradual shift of the coumarin-labeled amide-TBA from micelles to hydrogel, which precipitates out of the solution over time. The control experiment done in the absence of an enzyme did not show any degradation of DBA or aggregation of TBA (Figure S17), confirming the thermodynamic stability of this formulation.

Having showcased the transition of the amide-DBA and amide-TBA formulation from micelles to hydrogels, we proceeded to characterize the precipitated gel. Rheological measurement for this sample shows that the elastic modulus *G*′ was lower than the viscous modulus *G*″ (Figure 1H), which is typical for very soft physical hydrogels behaving predominantly as viscous liquids. Small-angle X-ray scattering (SAXS) was then used to characterize the hydrogels, and the obtained patterns showed that scattering was dominated by a single structure-factor correlation peak indicating the average distance between two cross-linking points of the hydrogel. This distance corresponds to 17 nm, which fits with the expected length of the TBA (Figure S33).^49^ The High-resolution scanning electron microscopy (HRSEM) image (Figure S32C) of the freeze-dried hydrogel shows the presence of a large number of interconnected pores, indicating the formation of hydrogels with super porous structure.

After characterizing the soft hydrogel that was formed, we set out to determine if it can undergo further enzymatic degradation and transform into soluble hydrophilic triblock polymers (Figure 1I). Following the extremely slow enzymatic degradation of TBA in its micellar state, we wished to expedite the enzymatic degradation process by using a 3.5-fold higher concentration of PGA, compared to the conditions that were used earlier for the induction of the transition from micelles to hydrogel. In addition, bovine serum albumin (BSA) was added to the sample containing the enzyme (as well as to the control sample without the enzyme) to shift the equilibrium toward the unimer state and hence expedite the enzymatic hydrolysis.^55^ Photos of the vials show the stability of the hydrogel in the absence of PGA and its full degradation into soluble hydrolyzed polymers in the presence of the enzyme (Figure 1I). To check the composition of the degraded gel, we took a small sample from the hydrolyzed polymer solution and analyzed it with HPLC. The same protocol was followed for the supernatant of the gel sample containing just BSA, aiming to determine if any degradation occurred under those conditions. The results of the HPLC analysis presented in Figure S34 show almost no degradation of the gel in the sample containing only BSA. In contrast, the sample containing BSA and the enzyme was fully hydrolyzed after 13 days, further confirming the complete degradation of TBA in the presence of PGA.

### Amide-DBA and Ester-TBA Formulation

**Figure 2:**
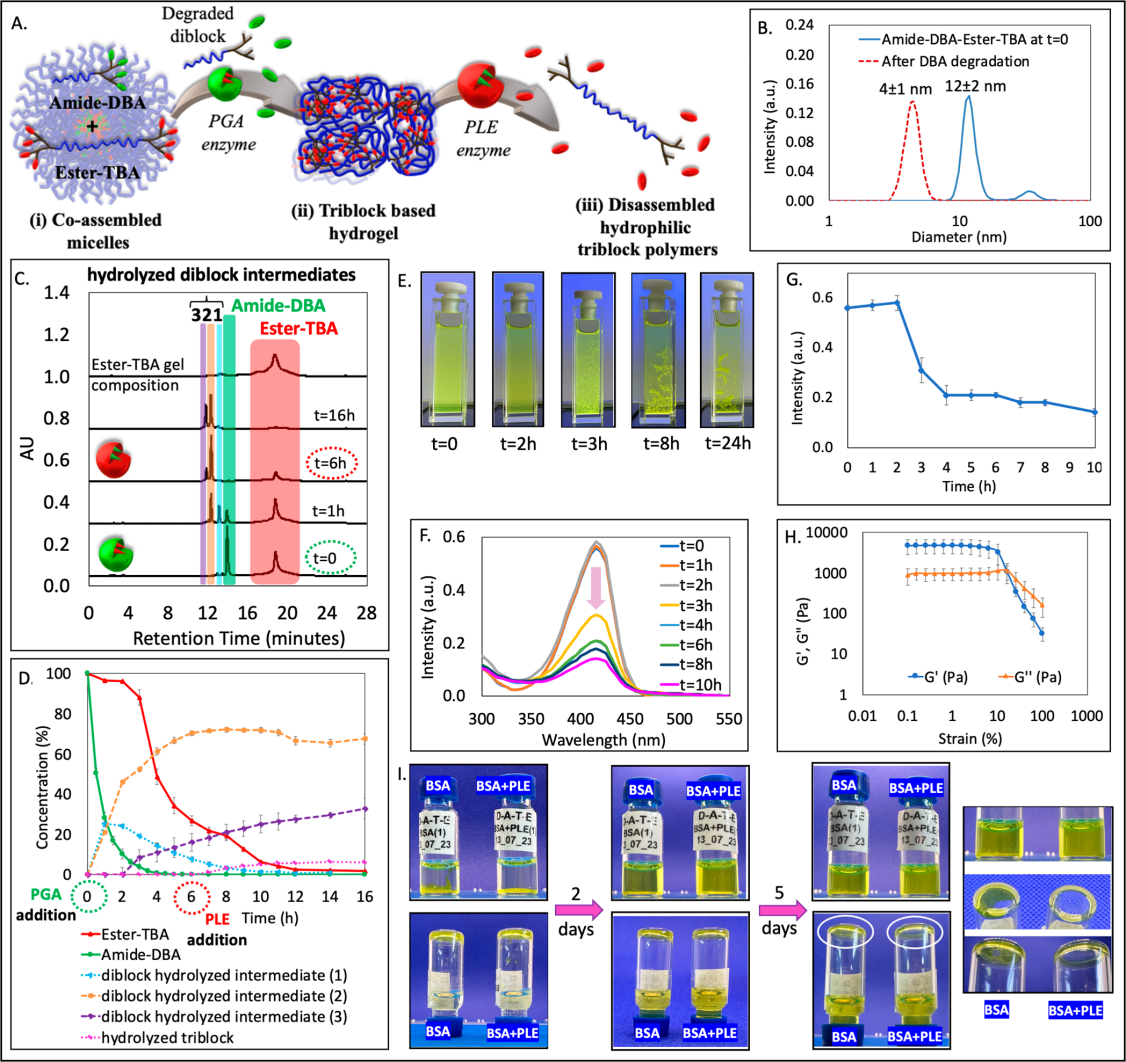
(A) Schematic illustration of the mesophase transition of amide-DBA and ester-TBA formulation from (i) coassembled mixed micelles into a (ii) hydrogel and finally to (iii) fully hydrolyzed polymers. (B) DLS of micelle solution before and after the addition of PGA, followed by PLE (at *t* = 6 h). (C) Overlay of HPLC chromatograms before and after the addition of PGA, followed by PLE (at *t* = 6 h), and the dissolved TBA-based hydrogel. (D) HPLC-based kinetic data. (E) Photos of the solution at different time points before and after the addition of PGA, followed by PLE (at *t* = 6 h). (F) Time-dependent absorbance spectra and (G) maximum intensity at 415 nm as a function of time. (H) Amplitude sweep test of the hydrogel at a constant frequency of 1 Hz. (I) Photos of the vials containing hydrogel with BSA or BSA and PLE over 7 days, indicating the transformation of hydrogel into hydrolyzed polymers in the presence of PLE (on the right—zoomed-in photos taken after 7 days).

After confirming the sequential mesophase transitions of the amide-DBA and amide-TBA formulation from micelles to hydrogel and then to degradable polymers in response to PGA, we proceeded to study more complex formulations that can respond to two different enzymes. Toward this, we mixed amide-DBA and ester-TBA at a 1:1 weight ratio and found this formulation to coassemble into micelles with a diameter of 12 nm as indicated by DLS and TEM (Figure 2B, solid line and S15D), and with a cmc of 5 μM (Figure S12). After confirming the formation of coassembled micelles, we proceeded to study the response of this micellar formulation to both amidase and esterase enzymes. We expected that upon incubating with the PGA enzyme first, the enzymatic degradation rate would follow a trend similar to that observed for the amide-DBA and amide-TBA formulation. As anticipated, HPLC analysis showed high selectivity of the PGA enzyme toward the degradation of amide-DBA (Figure 2C), which reached nearly 90% after 2 h, while ester-TBA remained nearly intact with approximately 4% degradation (Figure 2D). After 4 h, we observed a sudden drop in the ester-TBA concentration from 88 to 48%, which could be attributed to its aggregation into a hydrogel, as depicted in pictures taken at different time points (Figure 2E). After 6 h, we added an esterase (PLE), which can potentially cleave the ester end-groups of the ester-TBA. HPLC analyses showed a small amount (6%) of cleaved triblock (Figure S18), and the addition of PLE did not seem to affect the aggregation of ester-containing TBA into a hydrogel. DLS measurement conducted after 16 h did not show any presence of micelles, while structures with smaller diameters that can fit the hydrolyzed amide-DBA were observed (Figure 2B, dashed line). In parallel with the HPLC-based monitoring of the micelle to hydrogel transition, the absorbance spectra displayed a constant absorbance value for the initial 2 h, followed by a swift decrease in the subsequent hour (Figure 2F,G), aligning with the results obtained from the HPLC analysis. The control experiment done in the absence of an enzyme did not show any degradation of DBA or aggregation of TBA (Figure S23) confirming the thermodynamic stability of this formulation.

Subsequently, we proceeded to characterize the hydrogels formed from ester-TBA. The analysis of the composition of the formed hydrogel by dissolving it in acetonitrile (Figure 2C) revealed the expected presence of intact ester-TBA and also a very small amount (6%) of the hydrolyzed triblock. The HRSEM image for the freeze-dried sample of this hydrogel shows the presence of a porous structure, although less porous than the gel formed from amide-TBA (Figure S32D). In addition, SAXS measurement of the hydrogel showed a similar single structure-factor correlation peak, corresponding to 18 nm, which fits well the expected length of the ester-TBA (Figure S33). We then conducted rheological measurements of this hydrogel and observed that in the linear viscoelastic region of this sample, the elastic modulus *G*′ was higher than the viscous modulus *G*″ (Figure 2H), confirming the formation of a hydrogel. The pictures of the hydrogel in the rheometer (Figure S31) also show a difference in the appearance of the ester-TBA and amide-TBA hydrogels, which have different functional groups. These differences are most likely due to the relatively higher hydrophilicity of amide bonds in comparison with their ester equivalents as well as due to the aromatic nature of end-groups of the amide-TBA.

After characterizing the formed hydrogel, we studied the enzymatic degradation of the ester-TBA hydrogel. Similar to the degradation studies for the amide-TBA hydrogel, we accelerated the degradation by using a 3.5-fold higher PLE concentration and added BSA to shift the equilibrium. Photos in Figure 2I show the hydrogel’s stability with BSA and complete degradation in the presence of PLE. We further analyzed the composition of the solution of the degraded gel using HPLC and found that the sample containing BSA and PLE was fully hydrolyzed after 7days (Figure S35).

**Figure 3:**
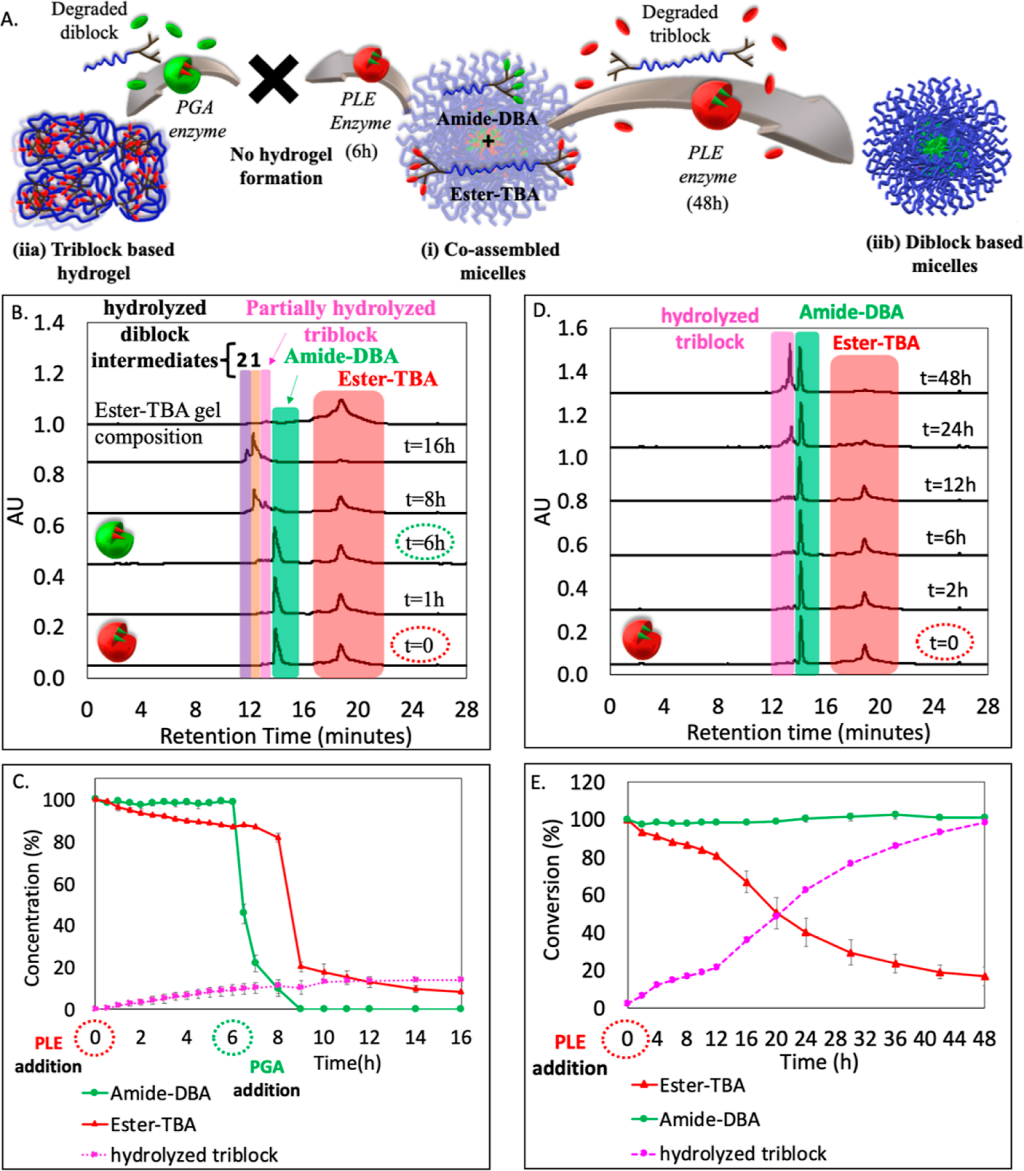
(A) Schematic illustration of the mesophase transition of amide-DBA and ester-TBA formulation: (i) coassembled mixed micelles transforming into (iia) a triblock-based hydrogel only upon addition of PGA (at *t* = 6 h) or (iib) diblock-based micelles upon prolonged incubation with PLE. (B) Overlay of HPLC chromatograms and (C) HPLC-based kinetic data for the addition of PLE at *t* = 0, followed by the addition of PGA at *t* = 6 h. (D) Overlay of HPLC chromatograms and (E) HPLC-based kinetic data upon incubating solely with PLE for an extended duration (48 h).

Next, we wished to investigate how the micellar formulation responds when we reverse the order of adding the enzymes and to study the effect of incubating the coassembled micelles with PLE first. HPLC analysis revealed slow degradation of ester-TBA (∼17%) and almost no degradation for amide-DBA during the initial 6 h of incubation with PLE (Figures 3B and S19). To gain a better understanding of the mesophase transition rate of this complex system, we opted to introduce the PGA enzyme after the initial 6 h incubation with PLE. Upon adding PGA, there was a sharp drop in the concentration of amide-DBA to 22%, accompanied by a subsequent drop in ester-TBA concentration (Figure 3C) and its simultaneous aggregation into a hydrogel (Figure S20).

We were then interested in exploring the behavior of this system when it was incubated solely with PLE for an extended duration. HPLC analysis showed that ester-TBA underwent slow degradation (∼82%) within 48 h of incubation with PLE, accompanied by the simultaneous formation of a new peak corresponding to the fully hydrolyzed triblock polymer (Figure 3D,E). In contrast, amide-DBA remained almost unaffected. DLS conducted at 48 h still showed the presence of micelles of around 11 nm (Figure S22A), and photos of the solution at this point showed that the solution remained clear (Figure S22B). These combined results show that PLE was selectively and slowly degrading ester-TBA leading to the formation of micelles composed solely from amide-DBA. These findings demonstrate that for this formulation to undergo a mesophase transition from micelle to hydrogel, it must first encounter an enzyme capable of breaking down DBA. Furthermore, it emphasizes the significance of having the DBA in order to preserve the TBA in its micellar form and allow its relatively faster enzymatic degradation through the unimer–micelle exchange when compared with the very slow degradation at the hydrogel mesophase.

### Ester-DBA and Amide-TBA Formulation

**Figure 4:**
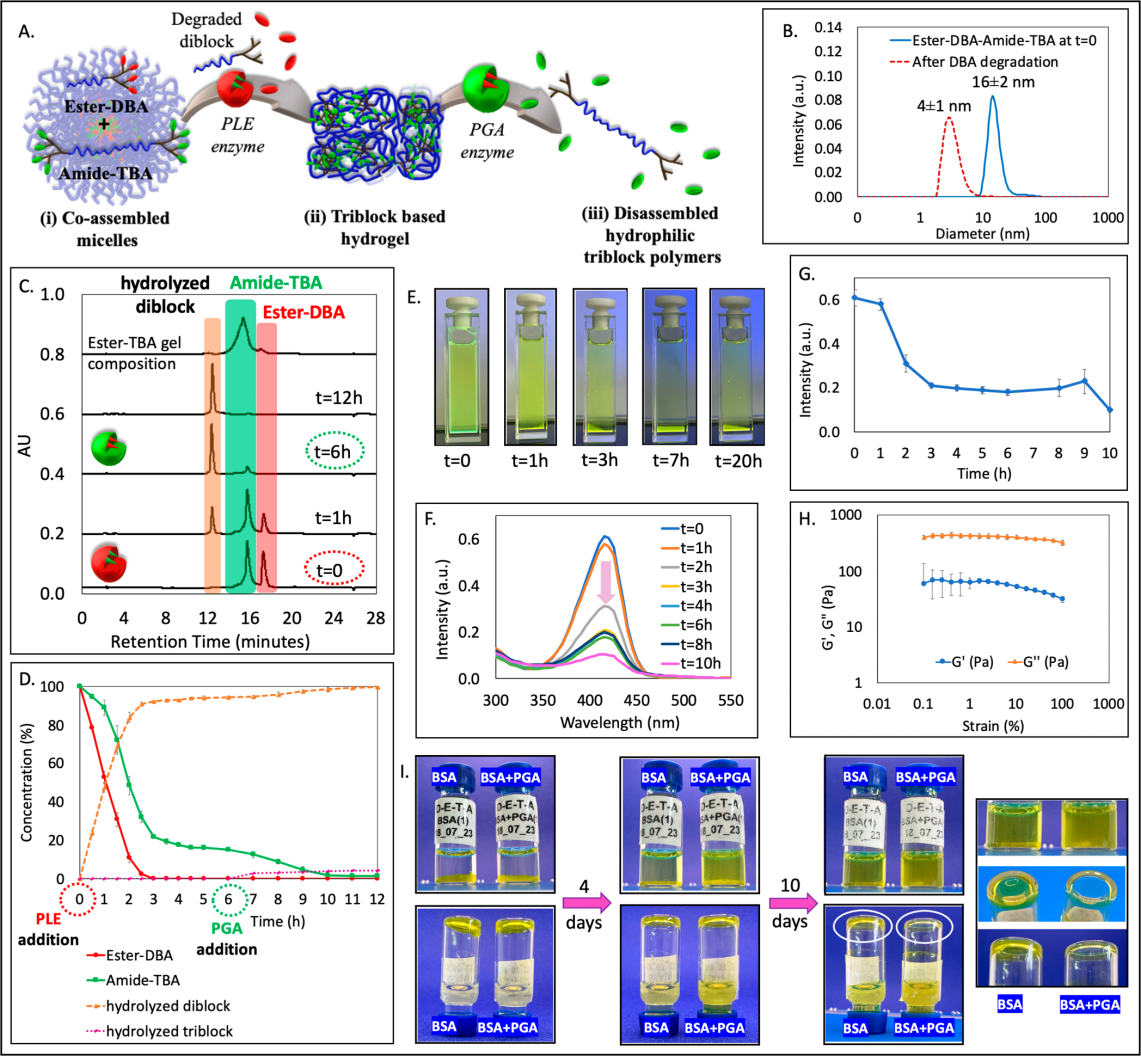
(A) Schematic illustration of the mesophase transition of ester-DBA and amide-TBA formulation from (i) coassembled mixed micelles into a (ii) hydrogel and finally to (iii) fully hydrolyzed polymers. (B) DLS of micelle solution before and after the addition of PLE, followed by PGA (at *t* = 6 h). (C) Overlay of HPLC chromatograms before and after the addition of PLE, followed by PGA (at *t* = 6 h), and the dissolved TBA-based hydrogel. (D) HPLC-based kinetic data. (E) Photos of the solution at different time points before and after the addition of PLE, followed by PGA (at *t* = 6 h). (F) Time-dependent absorbance spectra and (G) maximum intensity at 415 nm as a function of time. (H) Amplitude sweep test of the hydrogel at a constant frequency of 1 Hz. (I) Photos of the vials containing hydrogel with BSA or BSA and PGA over 14 days, indicating the transformation of hydrogel into hydrolyzed polymers in the presence of PGA (on the right—zoomed-in photos taken after 14 days).

After studying the different reaction cascades of the amide-DBA- and ester-TBA-based formulation and their dependence on the sequence of introducing the activating enzymes, we wished to study the reverse formulation, i.e. ester-DBA and amide-TBA. We mixed the two amphiphiles at a 1:1 weight ratio, and this formulation also showed micellar coassembly with a diameter of around 16 nm as indicated by DLS and TEM (Figure 4B, solid line, and S15E), and with a cmc of 4 μM (Figure S13), which are very similar to the values obtained for the amide-DBA- and ester-TBA-based micelles. After confirming the coassembly into mixed micelles, we conducted the enzymatic degradation of this formulation by first adding PLE, which could cleave the ester-DBA, followed by PGA, as depicted schematically in Figure 4A. HPLC analysis after adding PLE demonstrated the enzyme’s high selectivity for the ester-DBA, which reached nearly 100% degradation after 2.5 h (Figure 4C,D). For the amide-TBA, we observed a nearly simultaneous drop in its concentration in the first 2 h, and as the formation of cleaved triblock structures was not observed (Figure 4C) we can conclude that the reduction in amide-TBA is not a result of its enzymatic degradation. Instead, as observed for the other formulations, it can be attributed to its rapid aggregation into a hydrogel, which was noticeable already after just 1 h, as depicted in photos taken at different time points (Figure 4E). In parallel with the HPLC, the absorption measurements showed no change in the first hour, followed by a significant drop in the next 2 h, which aligned well with the results obtained from the HPLC analysis (Figure 4F,G). The addition of PGA after 6 h did not yield any significant change except for the emergence of a small amount of cleaved triblock (5%) as shown in Figure S24. DLS measurement conducted after the precipitation of the hydrogel was completed (at *t* = 12 h) and did not show any presence of micelles, and structures with diameter fitting the fully hydrolyzed hydrophilic diblock were observed (Figure 4B, dashed line). The composition of the formed hydrogels was analyzed by dissolving them in acetonitrile and was found to include the expected presence of amide-TBA with a minute amount (4%) of ester-DBA (Figure 4C). The control experiment done in the absence of an enzyme did not show any degradation of DBA or aggregation of TBA (Figure S29), confirming the thermodynamic stability of this formulation.

After demonstrating the transition of the ester-DBA and amide-TBA formulations from micelles to hydrogels, we characterized the resulting gel. Similar to the gel formed for the amide-DBA and amide-TBA, rheological measurements for this sample indicated that the elastic modulus *G*′ was lower than the viscous modulus *G*″ (Figure 4H), typical for very soft physical hydrogels that primarily exhibit viscous liquid behavior. In addition, similar to the other two formulations, the SAXS measurement of the hydrogel showed a single structure-factor correlation peak, corresponding to 18 nm, which fits well the expected length of the amide-TBA (Figure S33). Furthermore, the HRSEM image of the freeze-dried sample (Figure S32E) reveals numerous interconnected pores, supporting the development of a hydrogel with a porous structure.

Next, following the characterization of the formed hydrogel, we studied the enzymatic degradation of the ester-TBA hydrogel. Similar to the degradation studies conducted on the gel obtained from previous formulations, we enhanced the degradation process by employing a 3.5-fold higher PGA concentration and introduced BSA to shift the equilibrium. The images in Figure 4I depict the hydrogel’s stability in the presence of BSA and complete degradation when exposed to PGA along with BSA. As expected, the rheological properties of the hydrogel formed for this formulation and its degradation rate closely resemble those achieved with the amide-DBA and amide-TBA formulation, since the gel in both formulations is formed from amide-TBA.

**Figure 5:**
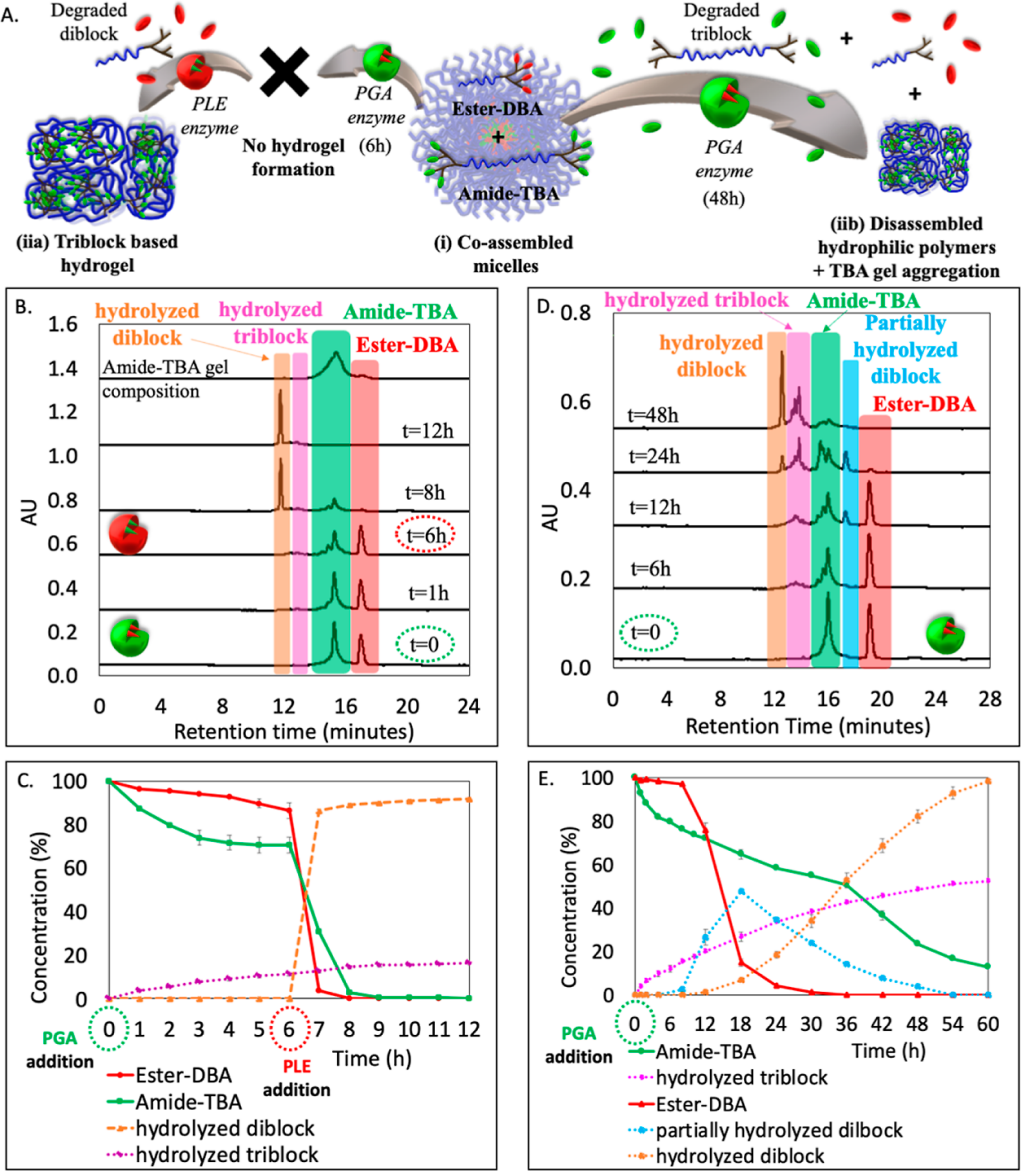
(A) Schematic illustration of the mesophase transition of ester-DBA and amide-TBA formulation: (i) coassembled mixed micelles transforming into (iia) a triblock-based hydrogel only upon addition of PLE (at *t* = 6 h) or (iib) disassembled hydrophilic polymers along with triblock-based aggregated gel upon prolong incubation with PGA. (B) Overlay of HPLC chromatograms and (C) HPLC-based kinetic data for the addition of PGA at *t* = 0, followed by the addition of PLE at *t* = 6 h. (D) Overlay of HPLC chromatograms and (E) HPLC-based kinetic data upon incubating solely with PGA for an extended duration (48 h).

Subsequently, we decided to reverse the order of adding the activating enzymes and began by adding PGA first. We observed a drop of around 30% in the concentration of amide-TBA and 14% for ester-DBA during the initial 6 h of incubation with PGA, followed by a parallel formation of partially cleaved triblock polymers. After 6 h, upon adding PLE to the same solution, we noticed a sharp decrease in the concentrations of both ester-DBA and amide-TBA, accompanied by the simultaneous formation of hydrolyzed diblock (Figure 5B,C). The pictures taken at different time points (Figure S26) show a distinct gel aggregation process after the addition of PLE to the sample. The chromatograms also did not indicate an extensive simultaneous formation of the cleaved triblock, implying that the disappearance of amide-TBA is not a result of enzymatic degradation but rather due to its aggregation into a hydrogel (Figure S25).

Similar to the previous formulation, we decided to explore the behavior of this system in the presence of a single enzyme, PGA, for an extended duration. The trend observed in HPLC-based kinetics was the same as before for the first 6 h. Notably, at later time points, we started observing the slow degradation of ester-DBA, followed by a sudden drop of around 85% in its concentration at 18 h (Figure 5D,E). We continued to monitor the degradation profile and observed that, in this case, PGA managed to degrade both ester-DBA and amide-TBA. After 36 h, when all the DBA got degraded, there was a drop in the concentration of amide-TBA, but no significant simultaneous formation of cleaved triblock was observed (Figure S27), indicating hydrogel formation. A picture of the vial taken at *t* = 48 h confirmed the presence of a small amount of aggregated TBA hydrogel (Figure S28A). To ensure that the observed hydrogel was formed by aggregation of TBA, we added acetonitrile to this sample and injected the obtained solution (∼1:1 ACN/PBS) into HPLC and observed the presence of amide-TBA (Figure S28B), as expected. DLS measurements conducted at the end of an enzymatic degradation experiment also showed the absence of micelles and the formation of 4 nm structures, which correlate with hydrolyzed diblock and triblock polymers (Figure S28C).

Looking at the obtained mesophase transitions for the two multienzyme responsive micellar formulations and their dependence on the sequence by which the activating enzymes were added provide important insights into the parameters that govern the ability to program the cascade response of such systems. The first key parameter is clearly the type of enzymatically cleavable end-group, which defines the reactivity and selectivity toward a specific enzyme. The other key parameter is the molecular architecture, as in both the mixed amide-DBA and ester-TBA and the ester-DBA and amide-TBA formulations, when the first stage of activation was conducted with an enzyme that can particularly degrade the DBAs end-groups, we observed the desired mesophase transition: from (i) micelles to (ii) triblock-based hydrogel. Moreover, upon the addition of an enzyme that can degrade the TBA end-groups, the obtained hydrogels could then undergo a second mesophase transition into (iii) fully hydrolyzed polymers. These cascade mesophase transitions corroborate our programming methodology which is based on using the DBA to initially stabilize the mixed micelles and then to induce the first mesophase transition upon its enzymatic degradation. Furthermore, even in cases in which the micellar formulations were first incubated for a few hours (<6 h) with the TBA degrading enzyme, no mesophase transition occurred, and upon the addition of the DBA cleaving enzyme, the systems could undergo the programmed mesophase transitions. This illustrates the key effect of molecular architecture and molecular weight in slowing down the rate of enzymatic degradation of the TBAs, making it significantly slower than their diblock counterparts, despite having identical dendritic blocks. It was astonishing to see the response of the two formulations when incubated with just the TBA degrading enzyme for longer durations (>8 h). Upon incubating the amide-DBA and ester-TBA mixed micelles with the PLE enzyme for an extended duration, the ester-TBA was slowly degraded by the enzyme, causing these mixed micelles to exhibit a transition from (i) coassembled micelles to (iib) diblock-based micelles (Figure 3A). This transition suggests that the stability of amide-DBA in the presence of the PLE enzyme, which cannot cleave the amide bonds in the DBA, plays a crucial role in maintaining the ester-TBA in a micellar form. Consequently, in contrast to the hydrogel mesophase, the micellar mesophase facilitates a more rapid exchange of TBA unimers and their subsequent degradation by the PLE enzyme. The slow degradation of the TBA in this formulation fits the small amount of degraded TBA, which we recently reported for the ester-DBA- and ester-TBA-based systems that showed around 10% degradation of TBA in the first 5 h under similar conditions.^50^ Hence, as mentioned above, the ability of the amide-DBA to maintain the ester-TBA in a micellar form in the presence of PLE allowed this slow degradation to proceed and reach the full enzymatic hydrolysis of the ester end-groups after 48 h.

In contrast, a very different result was observed when subjecting the ester-DBA and amide-TBA micellar formulations to extended incubation time with the PGA enzyme. This scenario encompassed a multifaceted response, progressing from (i) coassembled micelles to (iib) a blend of hydrolyzed triblock, hydrolyzed diblock, and TBA-based aggregated gel (Figure 5A). This complex and distinct outcome, in comparison with the incubation of the amide-DBA and ester-TBA micelles with PLE, is attributed to the lower stability of ester-DBA in the presence of the PGA enzyme. PGA, being less selective, could partially degrade the ester bonds in ester-DBA in parallel with the slow hydrolysis of the amide bonds in amide-TBA. The occurrence of both degradation processes simultaneously can explain the formation of both degraded DBA and TBA in the presence of PGA after long incubation times. Moreover, once the degradation of DBA reached a certain threshold, the remaining DBA could no longer stabilize the TBA in the micellar form, leading to its aggregation into a hydrogel mesophase. This mesophase transition of TBA slowed down its further degradation profile, eventually resulting in the observation of only 50% degradation out of the initial amount of TBA, while the remaining 50% aggregated into a hydrogel (Figure 5D,E).

In addition to understanding the effect of the type and sequence of added enzymes on the cascade of mesophase transitions, the comprehensive results also highlight the significant influence of the specific functional group of the TBA component of the formulation on the rheological properties of the resulting hydrogels. Regardless of the end-group, all three hydrogels yielded similar SAXS patterns dominated by a single structure-factor correlation peak, indicating an average distance between two cross-linking points of the hydrogel to be around 18 nm, which fits with the expected length of the TBAs. This indicates that the hydrogel is formed by the cross-linking of TBA-based micelles, in which the hydrophobic dendrons serve as cross-links and the hydrophilic PEG chains span the 3D network of the hydrogels. However, despite the similar formation of TBA-based hydrogels, the different end-groups resulted in hydrogels with distinct rheological properties. This is illustrated by the formation of a softer hydrogel with a more viscous-like behavior for the amide-TBA-containing formulations, while the ester-TBA-based hydrogel exhibited greater elastic behavior due to the difference in the degree of hydrophobicity of the two types of amphiphiles.

In addition to contributing to our understanding of the molecular parameters governing complex cascade mesophase transitions, this methodology can potentially be applied to the design of multifunctional drug delivery systems. These systems may be applied in micellar form, allowing for simple administration due to their liquid formulation. At the disease site, these micelles interact with the first enzymatic stimulus, degrading the DBA component and leading to the in situ formation of a biodegradable macroscopic hydrogel. This hydrogel can serve as a depot for prolonged drug release, undergoing slow enzymatic degradation by other (or similar) disease-associated enzymes. Further studies aimed at such applications and the development of additional molecular tools for programming cascades of mesophase transitions are currently ongoing.

## Conclusions

To summarize, we have incorporated different enzyme-responsive groups—namely, esterase and amidase—into both diblock and triblock amphiphiles. Through the utilization of these diverse enzyme-responsive structures, we successfully developed three distinct polymeric formulations (amide-DBA and amide-TBA, amide-DBA and ester-TBA, and ester-DBA and amide-TBA) by mixing the respective DBAs and TBAs in a 1:1 weight ratio. All of these formulations demonstrated cascade mesophase transitions: (i) micelles to (ii) hydrogel and finally to (iii) dissolved polymers upon initial exposure to an enzyme that specifically reacts with the end-groups present in the diblock architecture, followed by a second enzyme that can degrade the TBA-based hydrogel.

However, in formulations containing both types of enzyme-responsive groups, we noted intricate mesophase transitions when incubated with the TBA-cleaving enzyme for an extended duration depending on the specific type of enzyme and DBA in those formulations. The observed results demonstrate the ability to utilize multiple enzyme-responsive groups, which can respond to different enzymes, together with the polymeric architecture toward the design of formulations that can undergo programmed cascade mesophase transitions. These findings illustrate how the same polymeric system can respond very differently depending on the sequence and incubation time of the activating enzymes. The complex behavior resulting from the various scenarios can help us to understand the complexity of various biological macromolecules in nature. Additionally, it opens new opportunities for designing drug delivery systems capable of transforming into biodegradable drug depots at the target site, allowing for prolonged drug release.

## Supporting Information Available

Synthetic procedures, amphiphile and micelle characterization data, detailed experimental protocols, and control experiments (PDF)
