# Supplementary material for: Cascade Mesophase Transitions of Multi-enzyme Responsive Polymeric Formulations
Source: Biomacromolecules. 2024 May 22;25(6):3607–19. doi: 10.1021/acs.biomac.4c00221 (PMC11170936; doi:10.1021/acs.biomac.4c00221)
Supplement: Supplementary file 1 — bm4c00221_si_001.pdf [file bm4c00221_si_001.pdf]

Supporting Information

Cascade Mesophase Transitions of Multi-Enzyme Responsive Polymeric Formulations

Parul Rathee,[a,b,c] Nicole Edelstein-Pardo,[a,b,c] Gil Koren,[b,c,d] Roy Beck,[b,c,d] and Roey J. Amir[*, a,b,c,e]

[a]School of Chemistry, Faculty of Exact Sciences, Tel-Aviv University, Tel-Aviv 6997801, Israel

[b]The Center for Physics and Chemistry of Living Systems, Tel-Aviv University, Tel Aviv 6997801, Israel

[c]Center for Nanoscience and Nanotechnology, Tel-Aviv University, Tel Aviv 6997801, Israel

[d]School of Physics and Astronomy, Faculty of Exact Sciences, Tel-Aviv University, Tel-Aviv, 6997801, Israel

[e]ADAMA Center for Novel Delivery Systems in Crop Protection, Tel-Aviv University, Tel Aviv 6997801, Israel.

Contents

1. Instrumentation 3
2. Materials 3
3. Synthesis 4
   3.1 Synthesis of thiol functionalized hydrophobic end-groups 4
   3.2 Preparation of diblock amphiphiles (DBAs) 5
   3.3 Preparation of triblock amphiphiles (TBAs) 7
4. Characterization of DBAs and TBAs 10
4.1 HPLC measurements 10
4.2 Size exclusion chromatography (SEC) 11
4.3 Critical micelles concentration (CMC) measurements using Nile Red 12
4.4 Dynamic light scattering (DLS) 15
4.5 Transmission electron microscopy (TEM) 15
5. General sample preparation and enzymatic degradation studies 16
5.1 Enzymatic degradation experiments of amide-DBA and amide-TBA formulation 16
5.2 Enzymatic degradation experiments of amide-DBA and ester-TBA formulation 17
5.3 Enzymatic degradation experiments of ester-DBA and amide-TBA formulation 21
5.4 Absorption measurements 24
6. Characterization of the formed hydrogels and their further degradation 24
6.1 Rheology measurements 24
6.2 HRSEM measurements 25
6.3 Small Angle X-Ray Scattering (SAXS) 27
6.4 Hydrogel degradation 27
6.5 Analysis of the composition of the formed hydrogels 27

1. Instrumentation

HPLC: All measurements were recorded on a Waters Alliance e2695 separations module equipped with a Waters 2998 photodiode array detector. All solvents were purchased from Bio-Lab Chemicals and were used as received. All solvents are HPLC grade.

NMR: spectra were recorded on Bruker Avance III 400MHz/100MHz spectrometer. Chemical shifts are reported in ppm and referenced to the solvent. The molecular weights of the dendron-PEG-dendron tri-block copolymers were determined by comparison of the areas of the peaks corresponding to the PEG block (3.63 ppm) and the protons peaks of the dendrons.

SEC: All measurements were recorded on Viscotek SEC max by Malvern using refractive index detector and PEG standards (purchased from Sigma-Aldrich) were used for calibration. DMF (purchased from Sigma, HPLC grade) was used as the mobile phase. Columns (2 x PSS GRAM 1000Å) were used at a column temperature of 50°C.

DLS: All measurements were recorded on a Corduan Technology VASCO$^{\gamma}$ - particle size analyzer.

Absorbance Spectra: All measurements were recorded on a TECAN Infinite M200Pro device using quartz plate.

Rheometer: Rheological measurements were performed using a controlled- stress rheometer (AR-G2, TA instruments, USA). An 8 mm diameter flat-plate geometry with a crosshatched surface was used for the study.

SAXS: Measurements were performed using an in-house X-ray scattering system, with a Genix3D (Xenocs) low divergence Cu K_{α} radiation source (wavelength of λ = 1.54 Å) and scatter-less slits setup.

2. Materials

Poly(Ethylene Glycol) (10kDa), Poly(Ethylene Glycol) methyl ether (5kDa), allyl bromide (99%), 2,2-dimethoxy-2-phenylacetophenone (DMPA, 99%), Fmoc-Lys(Boc)-OH Novabiochem®, N,N'-Diisopropylcarbodiimide, Propargyl bromide, 80% solution in toluene, 4-nitrophenol (99.5%), 4-dimethylamino pyridine (DMAP), N,N'-dicyclohexylcarbodiimide (DCC, 99%), 2-mercaptoethanol, *Penicillin G Amidase from Escherichia coli* (PGA), *Porcine liver esterase* (PLE), Hexanoic acid, Triethylsilane, phenyl acetyl chloride, and Sephadex® LH20 were purchased from Sigma-Aldrich. Anhydrous potassium carbonate, and Trifluoroacetic acid (TFA) were purchased from Alfa Aesar. Potassium hydroxide, cystamine hydrochloride, Oxyma Pure Novabiochem® Triphenylmethyl chloride, Diisopropylethylamine (DIPEA), and bovine serum albumin (BSA, Probumin®) were purchased from Merck. 2-(1H-Benzotriazole-1-yl)-1,1,3,3-tetramethyluronium hexafluorophosphate was purchased from Chem-Impex. Piperidine, Silica Gel 60Å 0.040-0.063mm, sodium hydroxide, and all solvents were purchased from Bio-Lab and were used as received. Deuterated solvents for NMR were purchased from Cambridge Isotope Laboratories (CIL).

3. Synthesis

3.1 Synthesis of thiol functionalized hydrophobic end-groups.

Synthesis of porcine liver esterase (PLE) substrate (2-mercaptoethyl hexanoate):

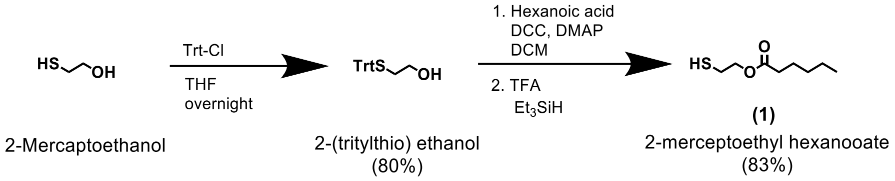

Scheme S1: Synthetic scheme for the preparation of ester containing thiol functionalized end-group (1).

<u>3-(tritylthio) ethanol:</u> Trityl chloride (17.1 g, 61.4 mmol, 1.2 eq.) was dissolved in THF (40 mL) and 3-mercaptoethanol (3.6 mL, 51.2 mmol, 1.0 eq.) was added to the flask. The reaction was stirred overnight at room temperature. Then, the solvent was evaporated in vacuum and the product was purified using flash silica chromatography (DCM: EtOAc 90:10). The product was obtained as a white solid in 80% yield (13.1 g).

<u>2-mercaptoethyl hexanoate (1):</u> 2-(tritylthio) ethanol (3.0 g, 9.4 mmol) and hexanoic acid (1.29 mL, 1.20 g, 10.3 mmol) were dissolved in 20 mL DCM. Then DCC (2.12 g, 10.3 mmol) was added followed by DMAP (0.34 g, 2.8 mmol). The reaction mixture was stirred at room temperature for about 4 hours and then the reaction mixture was filtered through a filter paper. To the filtrate 12 mL of TFA was added followed by triethylsilane (1.94 mL, 1.41 g, 12.17 mmol). The reaction was stirred for 30 minutes at ambient temperature and solvents were evaporated to dryness under vacuum. The product was purified using flash silica chromatography (Hex: DCM 1:1) (product was identified on TLC using $KMnO_4$). The product **(1)** was obtained as colorless oil in 83% yield (1.37 g).

*A previously reported synthetic protocol was followed[1] and the spectroscopic characterization correlated well with these reports.

Synthesis of penicillin G amidase (PGA) substrate (HS-PhAcAm):

Synthesis of disulfide-PhAcAm and HS-PhAcAm **(2)** was followed by a previous report[2] and the spectroscopic characterization correlated well with these reports.

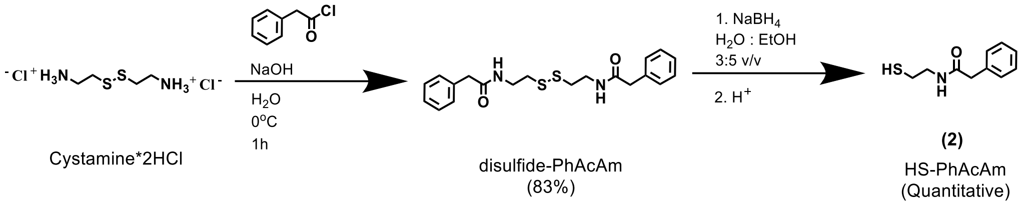

Scheme S2: Synthetic scheme for the preparation of amide containing thiol functionalized end-group (2).

3.2 Preparation of diblock amphiphiles (DBAs)

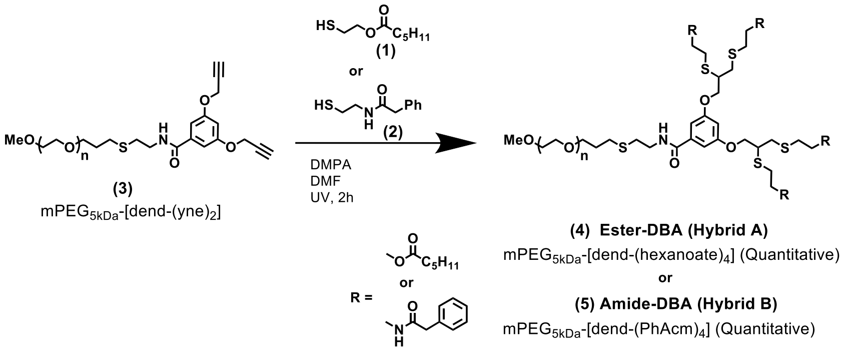

Scheme S3: Synthetic scheme for the preparation of ester-DBA (Hybrid A) and amide-DBA (Hybrid B).

* mPEG$_{5kDa}$-[dend-(yne)$_2$] **(3)** was synthesized as previously reported[3] and the spectroscopic characterization correlated well with these reports.

General procedure for thiol-yne reaction of mPEG$_{5kDa}$-[dende-(yne)$_2$] with thiol functionalized enzymatic end-groups:

mPEG$_{5kDa}$-[dend-(yne)$_2$] (1.0 g, 1 eq.) was dissolved in DMF (5 mL per 1 g). The chosen thiol [1.3 g, 40 eq. 2-mercaptoethyl hexanoate **(1)** or 1.5 g, 40 eq. HS-PhAcAm **(2)**] and DMPA (19.2 g, 0.4 eq.) were added. The solution was purged with nitrogen for 20 minutes and then stirred under UV light (365 nm) for 2 hours. Then, the reaction mixture was loaded as-is on a MeOH-based LH20 (Sephadex®) size exclusion column. Fractions that contained the product (identified by UV light and/or coloring with iodine) were unified, the organic solvents were evaporated to dryness and the white solid was dried under high vacuum.

mPEG$_{5kDa}$-[dend-(hexanoate)$_4$], ester-DBA (Hybrid A):

The product was obtained as white solid with quantitative yield (1.08 g).

^{1}H-NMR (400 MHz, $CDCl_3$): see following spectrum and assignments. SEC (DMF + 25 mM NH_4Ac): expected M_n= 6.0 kDa, experimental M_n = 5.9 kDa, $Đ_M$ = 1.05.

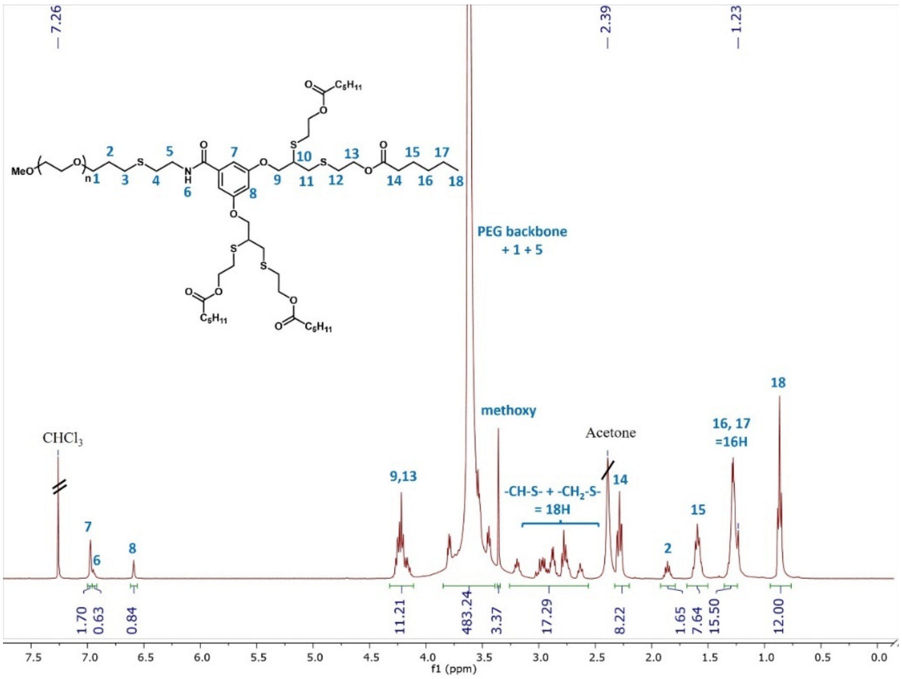

Figure S1: ^{1}H-NMR spectrum of ester-DBA {mPEG$_{5kDa}$-[dend-(hexanoate)$_4$], Hybrid A} in $CDCl_3$.

mPEG$_{5kDa}$-dend-(PhAcAm)$_4$], amide-DBA (Hybrid B):

The product was obtained as white solid with quantitative yield (1.08 g).

^{1}H-NMR (400 MHz, $CDCl_3$): see following spectrum and assignments. SEC (DMF + 25 mM NH_4Ac): expected M_n= 6.1 kDa, experimental M_n = 6.0 kDa, $Đ_M$ = 1.05.

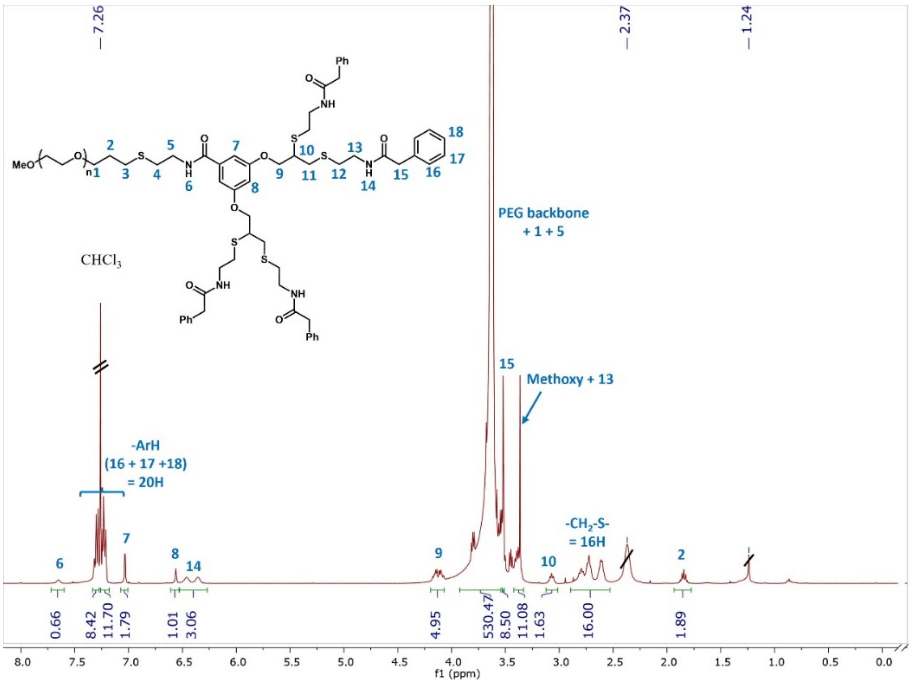

Figure S2: ^{1}H-NMR spectrum of amide-DBA {mPEG$_{5kDa}$-[dend-(PhAcAm)$_4$], Hybrid B} in $CDCl_3$.

3.3 Preparation of triblock amphiphiles (TBAs)

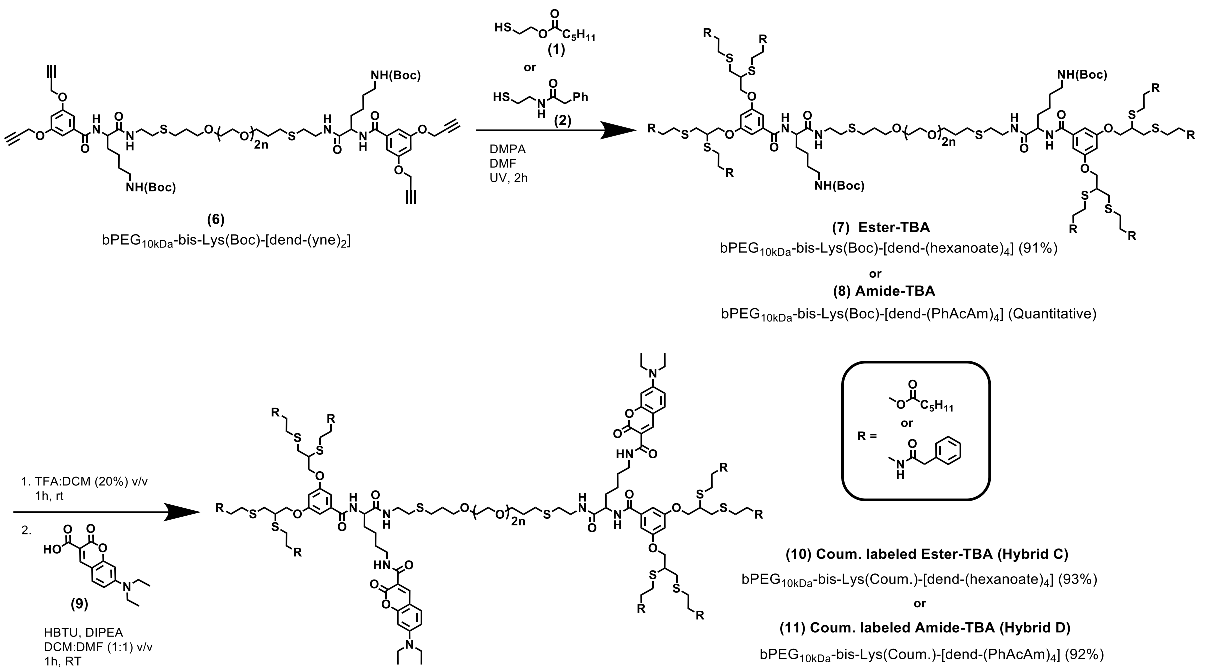

Scheme S4: Synthetic scheme for the preparation of coumarin labeled ester-TBA, $bPEG_{10kDa}$-bis-Lys(Coum.)-[dend-(hexanoate)$_4$] (Hybrid C) and coumarin labeled amide-TBA, $bPEG_{10kDa}$-bis-Lys(Coum.)-[dend-(PhAcAm)$_4$] (Hybrid D).

*$bPEG_{10kDa}$-bis-Lys(Boc)-[dend-(yne)$_2$] **(6)** was synthesized as previously reported[4] and the spectroscopic characterization correlated well with these reports.

General procedure for the synthesis of Boc-protected TBAs (compound 7 and 8):

$bPEG_{10kDa}$-bis-Lys(Boc)-[dend-(yne)$_2$] (500 mg, 1 eq.) was dissolved in DMF (5 mL per 1 g). The chosen thiol [634 mg, 80 eq. 2-mercaptoethyl hexanoate **(1)** or 703 mg, 80 eq. HS-PhAcAm **(2)**] and DMPA (9.2 g, 0.8 eq;) were added. The solution was purged with nitrogen for 20 minutes and then stirred under UV light (365 nm) for 2 hours. Then, the reaction mixture was loaded as-is on a MeOH-based LH20 (Sephadex®) size exclusion column. Fractions that contained the product (identified by UV light and/or coloring with iodine) were unified, the organic solvents were evaporated and further dried under a high vacuum. The products (compounds **7** and **8**) were obtained as white solids with a 91% yield (512 mg) and a quantitative yield (545 mg), respectively.

$bPEG_{10kDa}$-bis-Lys(Boc)-[dend-(hexanoate)$_4$], ester-TBA (7):

^{1}H-NMR (400 MHz, $CDCl_3$): see following spectrum and assignments. SEC (DMF + 25 mM NH_4Ac): expected M_n= 12.5 kDa, experimental M_n = 13.0 kDa, $Đ_M$ = 1.16.

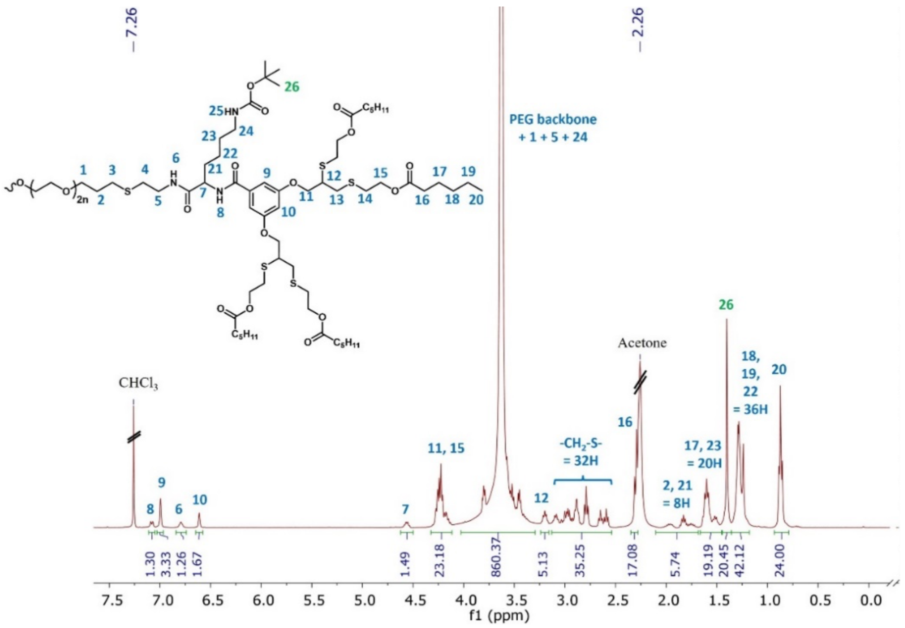

Figure S3: ^{1}H-NMR spectrum of ester-TBA {bPEG$_{10kDa}$-bis-Lys(Boc)-[dend-(hexanoate)$_4$]} in $CDCl_3$.

bPEG$_{10kDa}$-bis-Lys(Boc)-[dend-(PhAcAm)$_4$], amide-TBA (8):

^{1}H-NMR (400 MHz, $CDCl_3$): see following spectrum and assignments. SEC (DMF + 25 mM NH_4Ac): expected M_n= 12.7 kDa, experimental M_n = 13.7 kDa, $Đ_M$ = 1.16.

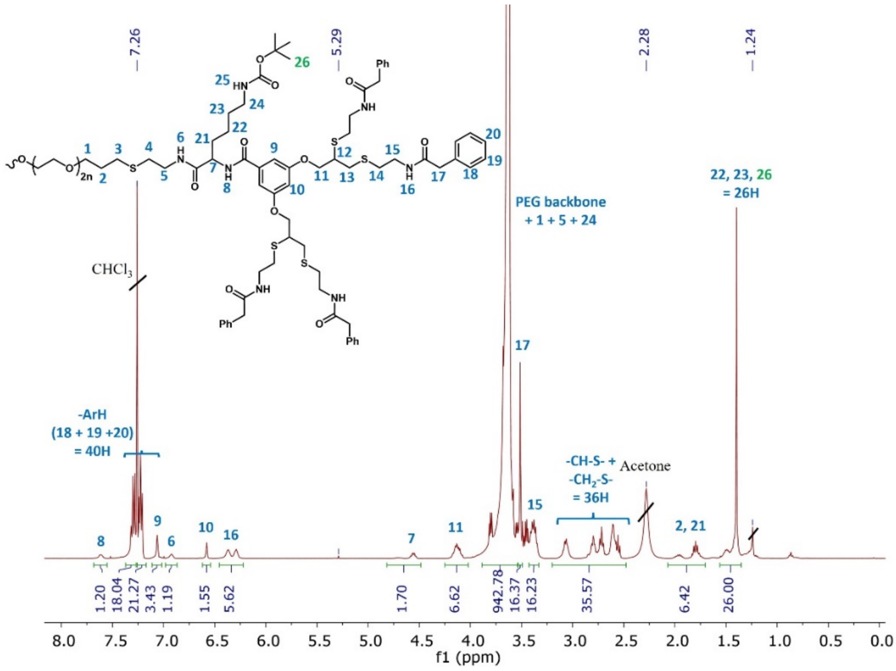

Figure S4: ^{1}H-NMR spectrum of amide-TBA {bPEG$_{10kDa}$-bis-Lys(Boc)-[dend-(PhAcAm)$_4$]} in $CDCl_3$.

General procedure for the synthesis of coumarin labeled TBAs :

200 mg (1eq.) of Boc protected TBAs (compound **7 or 8**) were dissolved in DCM (2 mL) and TFA (400 µL) {TFA:DCM, 1:5}. The mixture was allowed to stir for 1 hour and the reaction was monitored via HPLC. Once the Boc deprotection was confirmed by HPLC, DIPEA was added to the reaction mixture until fumes from it stopped coming out. After that MeOH-based LH20 SEC column was done to get rid of TFA.

Coumarin-acid (compound **9**, 26.5 mg, 6 eq.), HBTU (38.5 mg, 6 eq.) and DIPEA (65.6 mg, 88.5 µL, 30 eq.) were dissolved in a total volume of 2 mL DCM: DMF (2:1 v/v) in a 4 mL vial. The solution was stirred for 2 minutes and then it was added to the concentrated Boc deprotected polymer hybrid. The reaction mixture was allowed to stir for 2 hours and then loaded on a MeOH-based LH20 SEC column. The fractions containing the product were unified, and the MeOH was evaporated and further dried under high vacuum. The coumarin labeled TBAs products (Hybrid **C** and **D**) were obtained as yellow solids with a 93% yield (190 mg) and a 92% yield (188 mg), respectively.

bPEG$_{10kDa}$-bis-Lys(Coum.)-[dend-(hexanoate)$_4$], coumarin labeled ester-TBA (Hybrid C):

^{1}H-NMR (400 MHz, $CDCl_3$): see following spectrum and assignments. SEC (DMF + 25 mM NH_4Ac): expected M_n= 12.8 kDa, experimental M_n = 13.6 kDa, $Đ_M$ = 1.19.

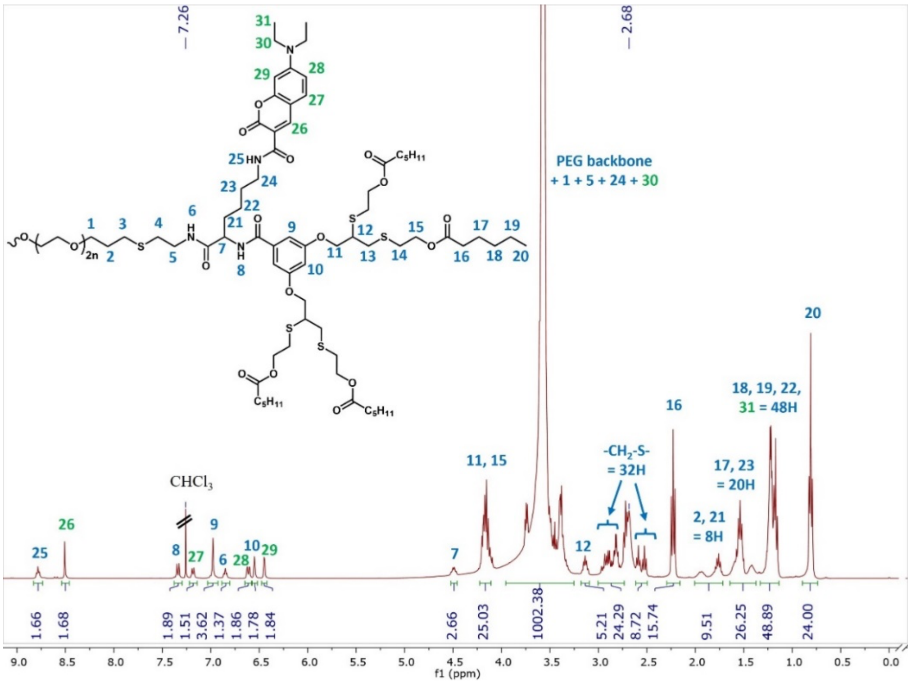

Figure S5: ^{1}H-NMR spectrum of coumarin labeled ester-TBA {bPEG$_{10kDa}$-bis-Lys(Coum.)-[dend-(hexanoate)$_4$], Hybrid C} in $CDCl_3$.

bPEG$_{10kDa}$-bis-Lys(Coum.)-[dend-(PhAcAm)$_4$], coumarin labeled amide-TBA (Hybrid D):

^{1}H-NMR (400 MHz, $CDCl_3$): see following spectrum and assignments. SEC (DMF + 25 mM NH_4Ac): expected M_n= 13.0 kDa, experimental M_n = 14.6 kDa, $Đ_M$ = 1.16.

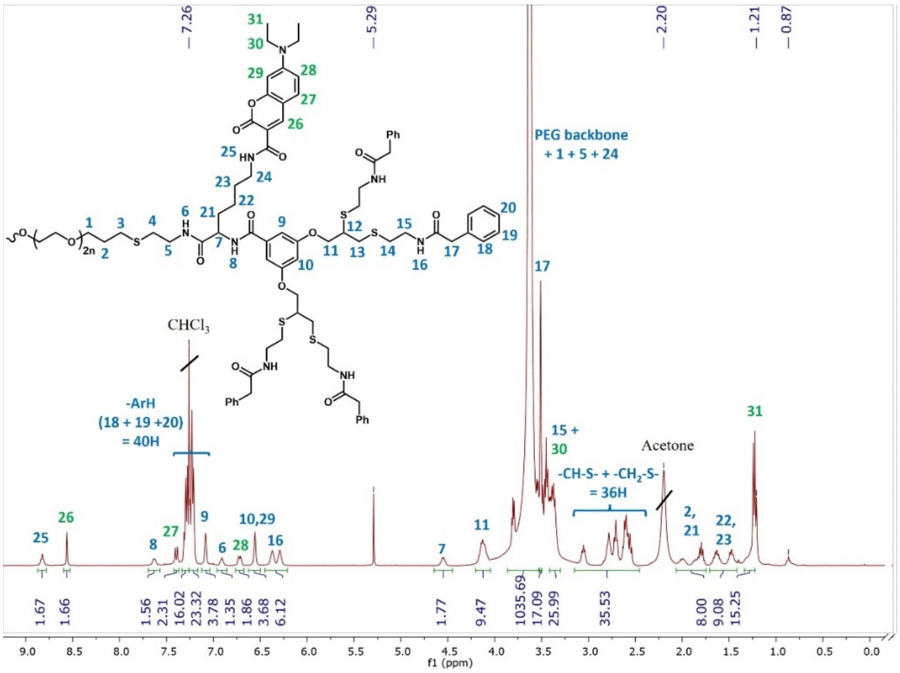

Figure S6: ^{1}H-NMR spectrum of coumarin labeled amide-TBA {bPEG$_{10kDa}$-bis-Lys(Coum.)-[dend-(PhAcAm)$_4$], Hybrid D} in $CDCl_3$.

4. Characterization of DBAs and TBAs

4.1 HPLC measurements

Instrument: Waters Alliance e2695

Column: Aeris WIDEPORE, C4, 3.6 µm, 150 x 4.6 mm

Column temperature: 37 °C

Sample temperature: 37 °C

Solution A: 0.1% $HClO_4$, H_2O: ACN 95:5v/v

Solution B: 0.1% $HClO_4$, H_2O: ACN 5:95v/v

Solution C: ACN

Flow rate: 1.0 mL/min

Gradient program for 30-minute injection:

Time (minutes)	Sol. A (%)	Sol. B (%)	Sol. C (%)
**0.0**	95	0	5
**1.0**	95	0	5
**20.0**	0	95	5
**23.0**	0	95	5
**23.1**	95	0	5
**30.0**	95	0	5

Injection Volume: 30 µL

Seal wash: H_2O: MeOH 90:10v/v

Needle wash: MeOH

Detector: Waters 2998 photodiode array detector

Sampling rate: 2 points/sec

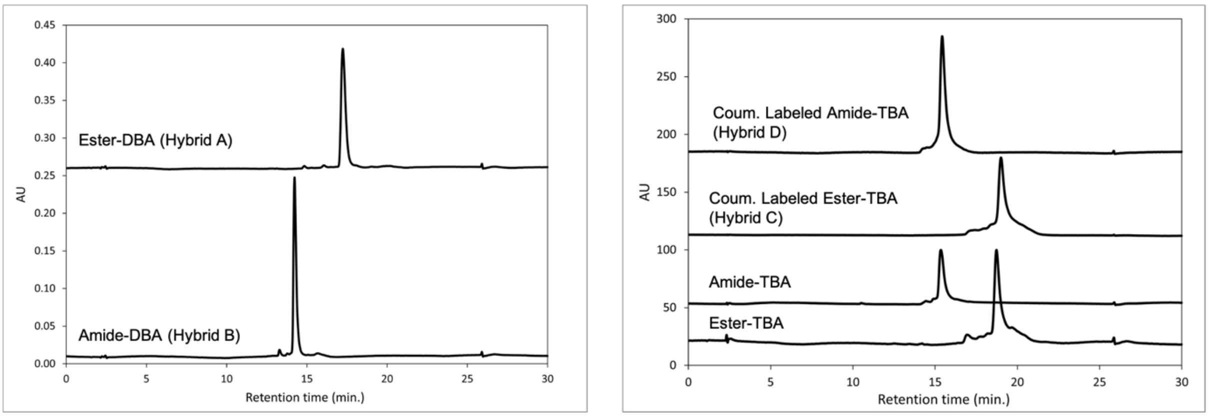

Figure S7: HPLC overlay of DBAs (left) and TBAs (right).

4.2 Size exclusion chromatography (SEC)

Instrument: Malvern Viscotek GPCmax

Columns: 2xPSS GRAM 1000 Å

Columns temperature: 50 °C

Flow rate: 0.5 mL/min

Injection time: 60 min

Injection volume: 50 µL from a 10 mg/mL sample

Diluent + mobile phase: DMF + 25 mM NH_4Ac

Needle wash: DMF

Detector: Viscotek VE3580 RI detector

Sample preparation: The amphiphiles were directly dissolved in the diluent to give a final concentration of 10 mg/mL and filtered through a 0.22 µm PTFE syringe filter.

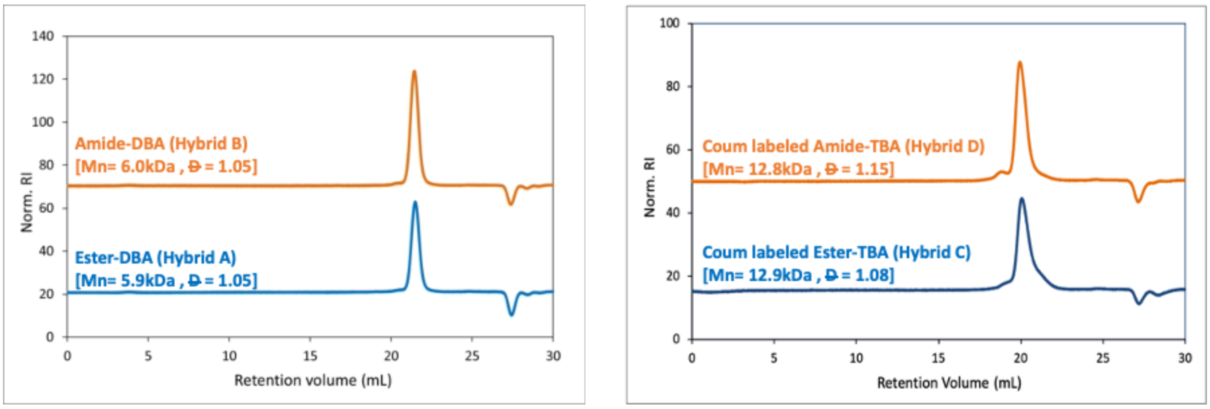

Figure S8: SEC overlay of final hybrids of DBAs (left) and TBAs (right).

4.3 Critical micelles concentration (CMC) measurements using Nile Red

General procedure of measurement:

Preparation of diluent:

Nile Red stock solution (0.80 mg/mL in ethanol) was diluted into a phosphate buffer saline (137 mM NaCl, 10 mM phosphate, 2.7 mM KCl; pH 7.4) to afford a final concentration of 1.25 µM.

Preparation and measurement of samples:

The PEG-dendron amphiphiles were directly dissolved in the diluent to give a final concentration of 500 µM. The solution was vortexed vigorously until the amphiphiles were completely dissolved and further sonicated for 15 minutes in an ultrasonic bath. The solutions were consecutively diluted by a factor of 1.5 with the diluent to afford a series of 24 samples for each amphiphile. 150 µL of each sample was loaded onto a 96-well plate and a fluorescence emission scan was performed for each well. To determine the hybrid's CMC – the maximum emission of Nile Red (at about 640 nm) was plotted as a function of the amphiphile's concentration. This procedure was repeated thrice for each amphiphile, and the mean value is reported as the CMC value and the standard deviation as measurement error.

Instrument method:

Instrument: TECAN Infinite M200Pro

Excitation: 550 nm

Emission intensity scan: 580-800 nm

Step: 5 nm

Number of flashes: 4

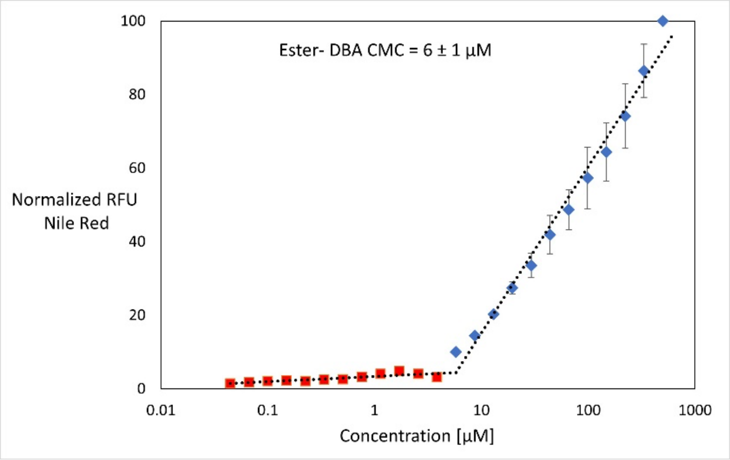

Figure S9: CMC measurement of ester-DBA, mPEG$_{5kDa}$-[dend-(hexanoate)$_4$].

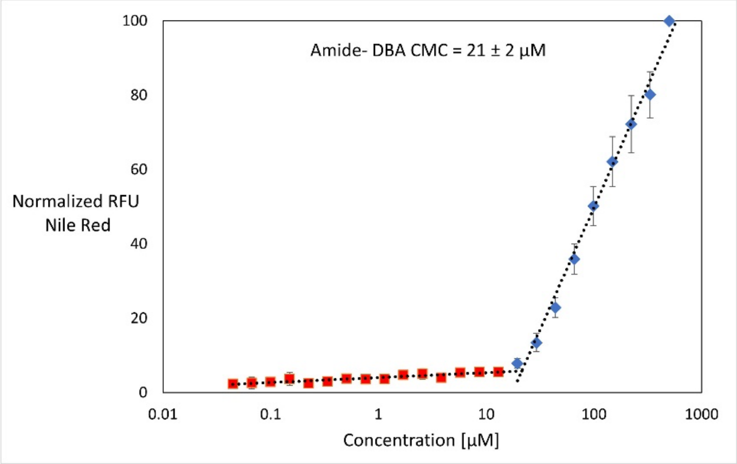

Figure S10: CMC measurement of amide-DBA, mPEG$_{5kDa}$-[dend-(PhAcAm)$_4$].

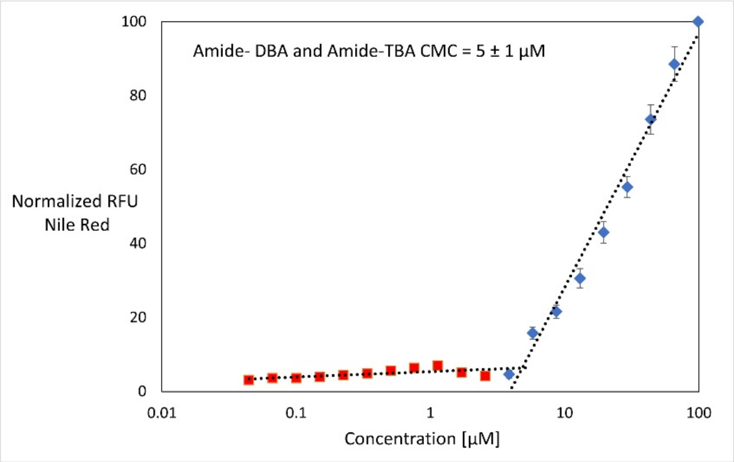

Figure S11: CMC measurement of amide-DBA and amide-TBA formulation.

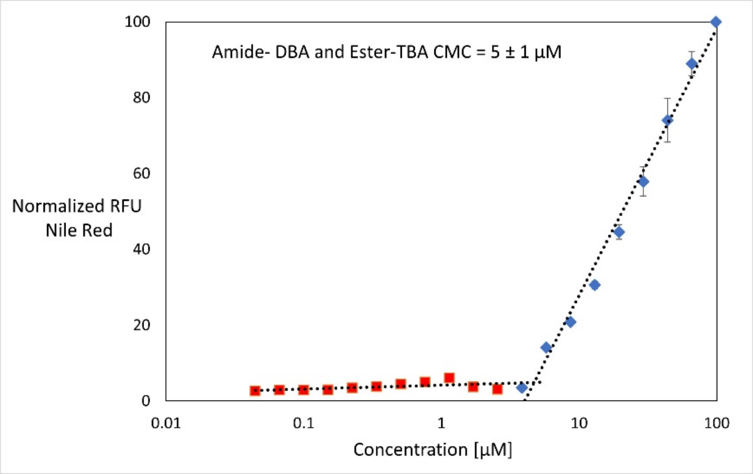

Figure S12: CMC measurement of amide-DBA and ester-TBA formulation.

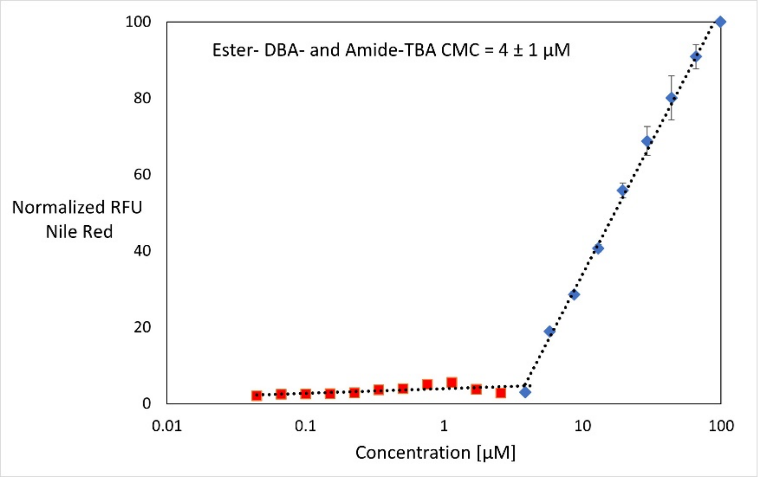

Figure S13: CMC measurement of ester-DBA and amide-TBA formulation.

4.4 Dynamic light scattering (DLS)

General sample preparation:

The PEG-dendron hybrids were directly dissolved in phosphate buffer (pH 7.4) to afford a final concentration of 5 mg/mL. The solution was vortexed and sonicated until a clear solution was obtained. Then, the solution was filtered through 0.22 µm nylon syringe filter and micellar diameters (D_H) were recorded in nm as a function of intensity.

A micellar solution (1:1 DBA: TBA weight ratio) was prepared by mixing 5 mg of each DBA (Hybrid **A or B**) and TBA (Hybrid **C or D**) in 1 mL PBS giving a total polymers concentration of 10 mg/mL to prepare three different co-assembled mixed micellar formulations. Vials were vortexed until full solubility was obtained, and then the solutions were sonicated for 15 minutes and filtered through a 0.22 µm nylon syringe filter. Measurements were performed at t = 0 before the addition of the required enzyme.

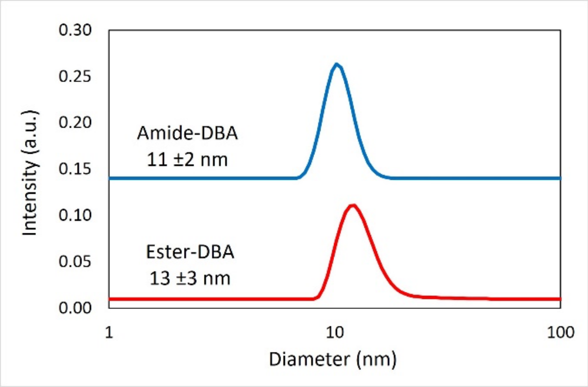

Figure S14: DLS size measurements overlay and analyzed D_H values of DBAs.

4.5 Transmission electron microscopy (TEM)

General sample preparation:

A micellar solution of DBA was prepared by mixing 5 mg of DBA (Ester-DBA or Amide-DBA) in 1 mL PBS to give a polymer concentration of 5 mg/mL.

A micellar solution for three different co-assembled micellar formulations (1:1 DBA: TBA weight ratio) was prepared by mixing 5 mg of each respective DBA and TBA in 1 mL PBS, giving a total polymer concentration of 10 mg/mL. Vials were vortexed until full solubility was obtained, and then the solutions were sonicated for 15 minutes. 30 µL of the solution was dropped onto carbon-coated copper grids. The excessive solvent of the droplet was wiped away using filter paper and the sample grids were left to dry in air at RT. Then, grids were inspected in a transmission electron microscope (TEM), operated at 2000 kV.

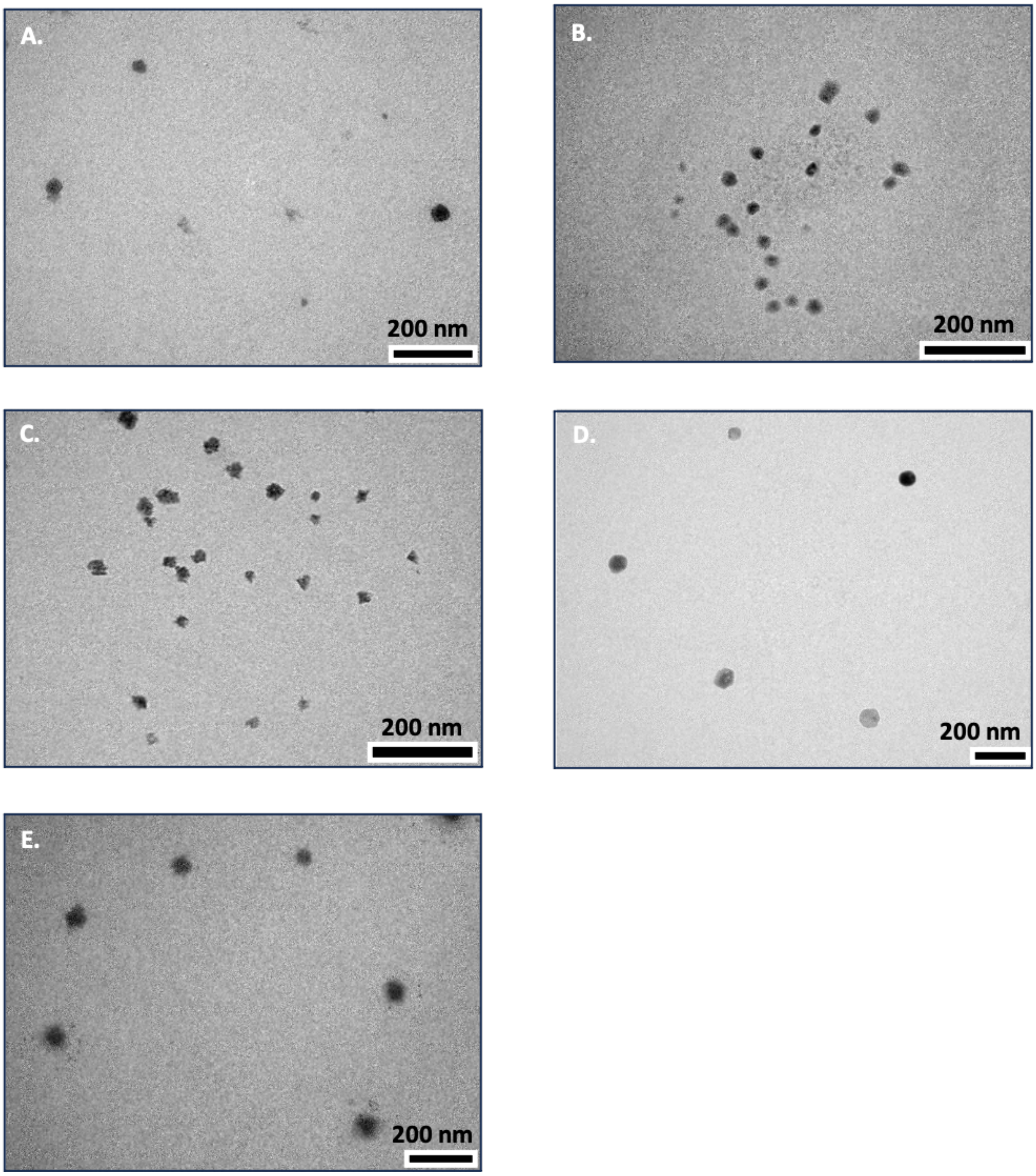

Figure S15: TEM images for micelles formed from DBA at a concentration of 5 mg/mL. (A) Ester-DBA and (B) Amide-DBA. TEM images for co-assembled micelles (t = 0) at a total polymer concentration of 10 mg/mL in 1:1 weight ratio. (C) Amide-DBA and amide-TBA, (D) amide-DBA and ester-TBA, and (E) ester-DBA and amide-TBA.

5. General sample preparation and enzymatic degradation studies

5.1 Enzymatic degradation experiments of amide-DBA and amide-TBA formulation

The micellar formulation was prepared by mixing 5 mg of each amide-DBA (compound 5) and amide-TBA (compound 11) in a 1:1 weight ratio. PGA was added to yield a final concentration

of 0.52 U/mL and degradation was followed at 37 °C by monitoring the area under the peak of the parent DBA, TBA and hydrolyzed polymer by HPLC at 297 nm. Each experiment was conducted thrice; the reported values in each time point are the mean value, and the standard deviation is the error (results shown in Figures 1C and 1D of the main text).

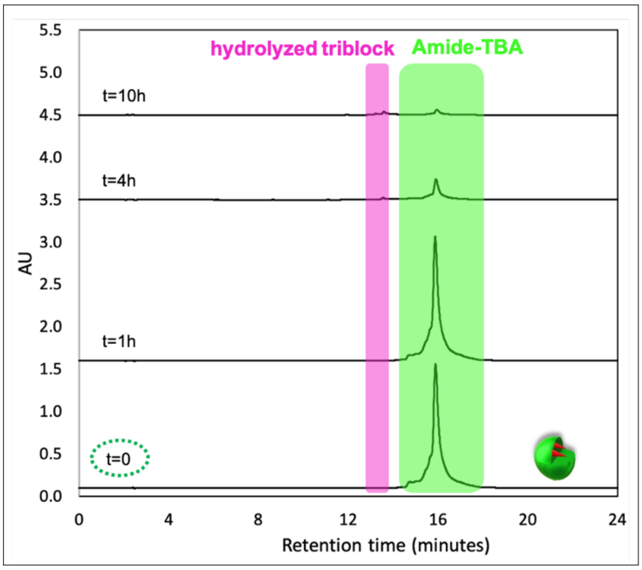

Figure S16: Overlay of HPLC chromatograms at 423 nm before and after the addition of PGA.

To monitor the thermodynamic stability of this mixed micellar solution, control experiments were done without adding an enzyme. The degradation was followed at 37 °C by monitoring the area under the peak of the parent amphiphile by HPLC at 297 nm. A picture of the solution taken after 12 hours did not show any aggregation of amide-TBA, indicating the stability of this formulation in the absence of an enzyme.

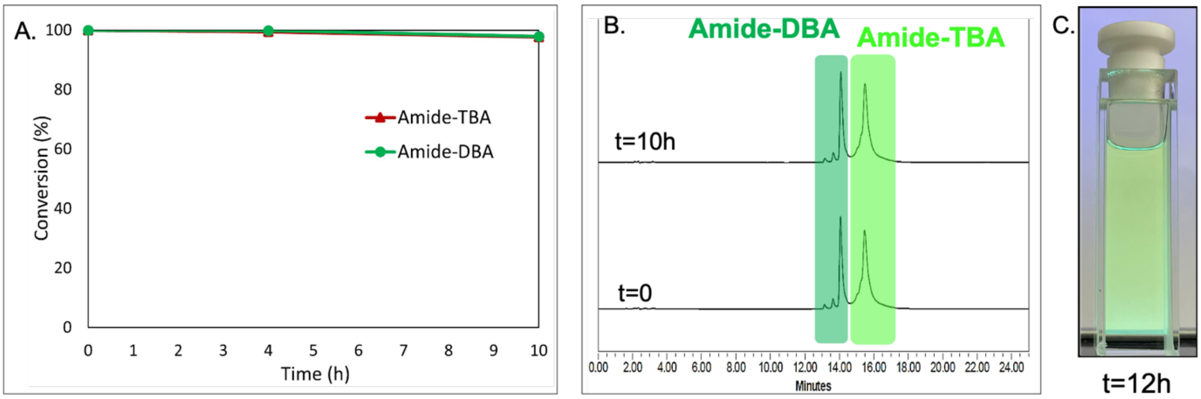

Figure S17: Amide-DBA and amide-TBA formulation in the absence of an enzyme (a) HPLC-based kinetics, (b) HPLC overlay, and (c) Photo of the solution.

5.2 Enzymatic degradation experiments of amide-DBA and ester-TBA formulation

The micellar formulation was prepared by mixing 5 mg of each amide-DBA (compound 5) and ester-TBA (compound 10) in a 1:1 weight ratio. The enzymatic degradation of this formulation was monitored through two distinct pathways: by altering the sequence in which we incubate the micellar solution with an enzyme, as illustrated schematically in Figures 2A and 3A of the main text.

For the first approach, PGA was added to yield a final concentration of 0.52 U/mL and degradation was followed at 37 °C by monitoring the area under the peak of the parent DBA, TBA and hydrolyzed polymer by HPLC at 297 nm. At t = 6 hours, PLE was added to yield a final concentration of 10 U/mL and continued following the degradation via HPLC. Each experiment was conducted thrice; the reported values in each time point are the mean value, and the standard deviation is the error (results shown in Figures 2C and 2D of the main text).

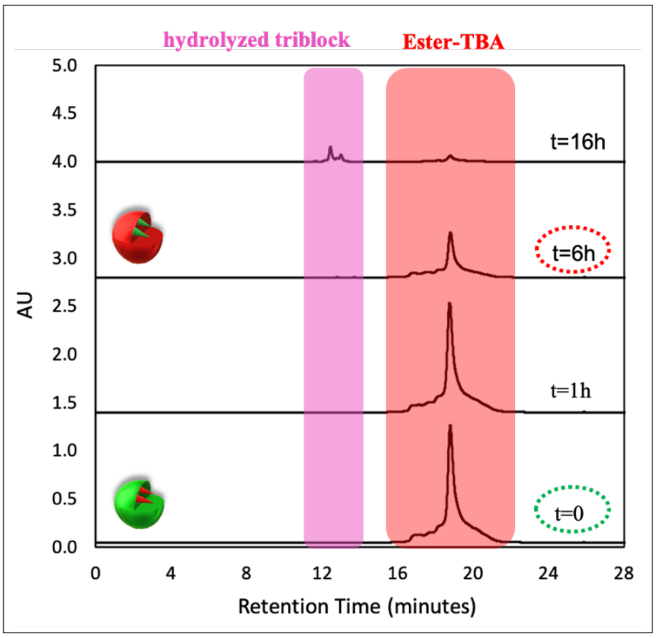

Figure S18: Overlay of HPLC chromatograms at 423 nm before and after the addition of PGA, followed by PLE (at t = 6 hours).

For the second pathway, we incubated the formulation with PLE at t = 0 and PGA was added at t = 6 hours while maintaining their concentrations at the same levels as before. The degradation was followed by HPLC at 297 nm, and each experiment was conducted thrice (Figures 3B and 3C of the main text). Pictures of the solution taken at different time points (Figure S20) did not reveal any aggregation of ester-TBA when incubated with PLE enzyme alone (up to t = 6 hours). However, at t = 8 hours, we observed some gel aggregation, occurring 2 hours after the same solution was incubated with the PGA enzyme.

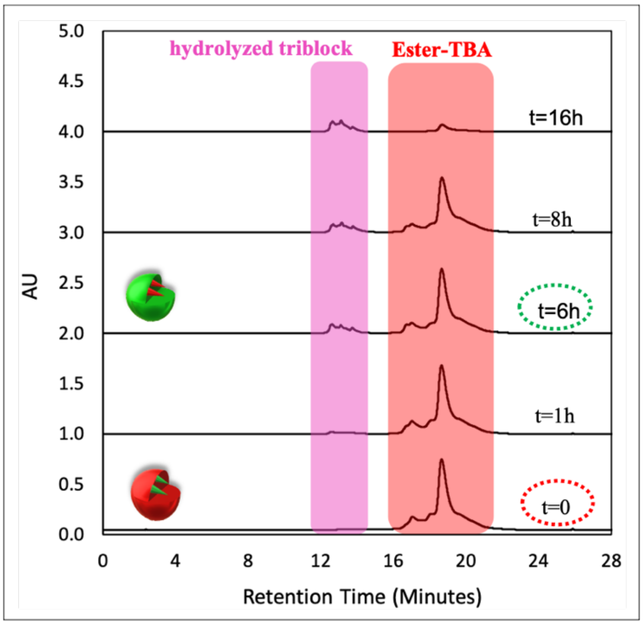

Figure S19: Overlay of HPLC chromatograms at 423 nm before and after the addition of PLE, followed by PGA (at t = 6 hours).

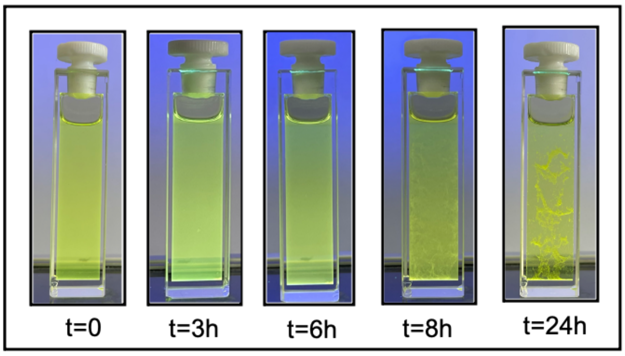

Figure S20: Photos of the amide-DBA and ester-TBA formulation at different time points before and after the addition of PLE, followed by PGA (at t = 6 hours).

We also monitored the response of this formulation upon activation with PLE only. The reported results are demonstrated in Figures 3D and 3E of the main text. The DLS measurement done after 48 hours of incubation with PLE enzyme showed the formation of micelles with a diameter of around 11 nm. Pictures of the solution taken at different time points (Figure S22) also did not reveal any aggregation of ester-TBA.

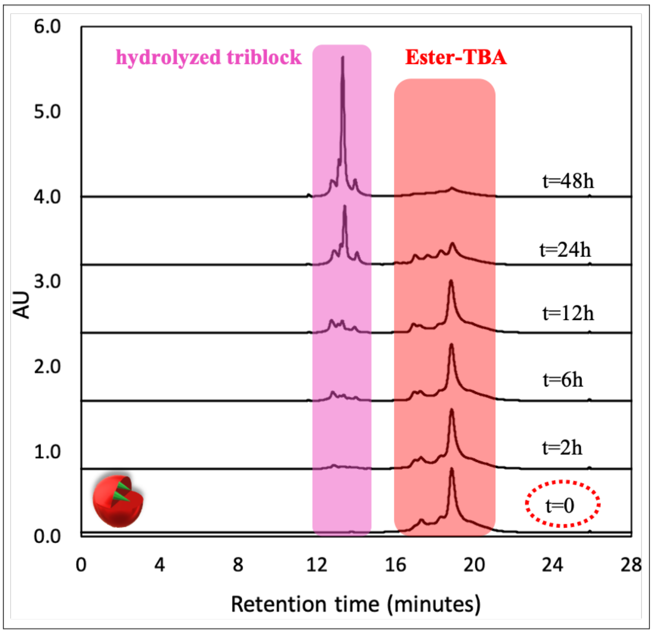

Figure S21: Overlay of HPLC chromatograms at 423 nm upon incubating solely with PLE for an extended duration (48 hours).

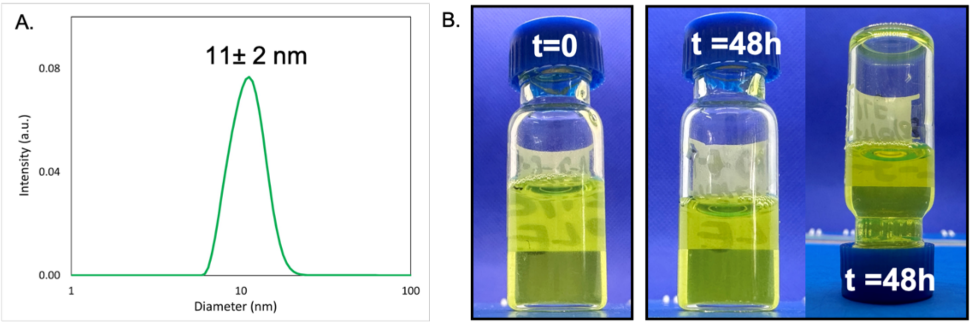

Figure S22: Amide-DBA and ester-TBA formulation upon incubation with PLE. (A) DLS measurement after 48 hours and (B) Pictures of the solution at t = 0 and t = 48 hours of incubation with PLE.

To monitor the thermodynamic stability of the mixed micelles obtained from amide-DBA and ester-TBA formulation, control experiments were done without adding an enzyme. The degradation was followed at 37 °C by monitoring the area under the peak of the parent amphiphile by HPLC at 297 nm. A picture of the solution taken after 24 hours indicates the stability of this formulation in the absence of an enzyme.

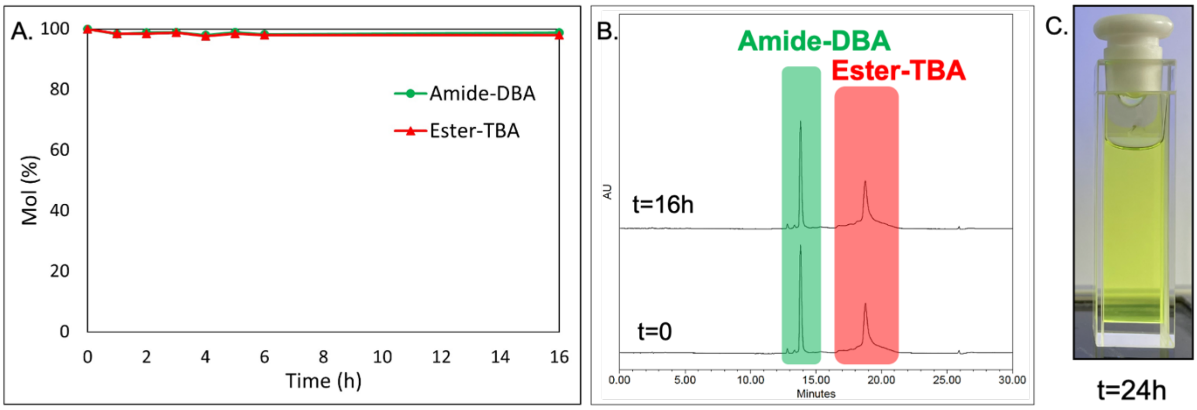

Figure S23: Amide-DBA and ester-TBA formulation in the absence of an enzyme (a) HPLC-based kinetics, (b) HPLC overlay, and (c) Photo of the solution.

5.3 Enzymatic degradation experiments of ester-DBA and amide-TBA formulation

This micellar formulation was prepared by mixing 5 mg of each ester-DBA (compound 4) and amide-TBA (compound 11) in a 1:1 weight ratio. The enzymatic degradation for this formulation was also monitored through two distinct pathways: by altering the sequence in which we incubate the micellar solution with an enzyme, as illustrated schematically in Figures 4A and 5A of the main text.

For the first approach, PLE was added to yield a final concentration of 3.5 U/mL and degradation was followed at 37 °C by monitoring the area under the peak of the parent DBA, TBA and hydrolyzed polymer by HPLC at 297 nm. At t = 6 hours, PGA was added to yield a final concentration of 2.62 U/mL and continued following the degradation via HPLC. Each experiment was conducted thrice; the reported values in each time point are the mean value, and the standard deviation is the error (Figures 4C and 4D of the main text). A lower concentration of PLE was chosen here to get more time points during the degradation process.

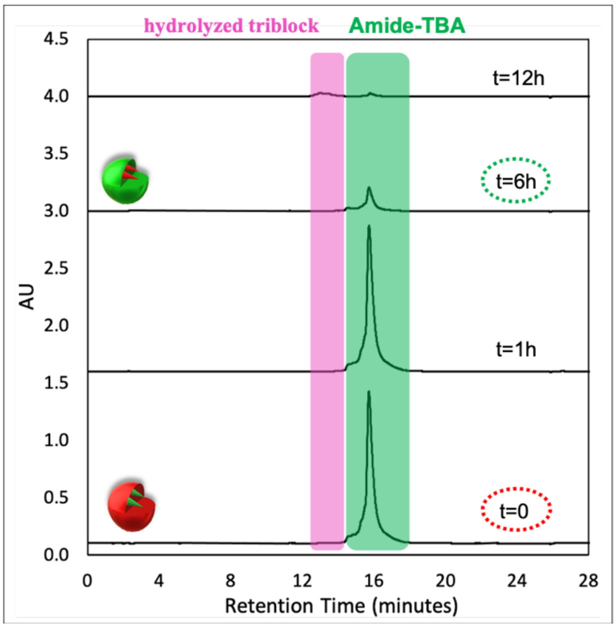

Figure S24: Overlay of HPLC chromatograms at 423 nm before and after the addition of PLE, followed by PGA (at t = 6 hours).

For the second pathway, the enzyme was incubated at different time points (PGA at t = 0 and PLE at t = 6 hours) while maintaining their concentrations at the same levels as before. The degradation was followed by HPLC, and each experiment was conducted thrice. The reported values in each time point are the mean value, and the standard deviation is the error (as shown in Figures 5B and 5C of the main text). Pictures of the solution taken at different time points did not reveal any considerable aggregation of amide-TBA when incubated with PGA enzyme alone (up to t = 6 hours). However, at t = 7 hours, a distinct gel aggregation process was observed, 1 hour after the same solution was incubated with the PLE enzyme (Figure S26).

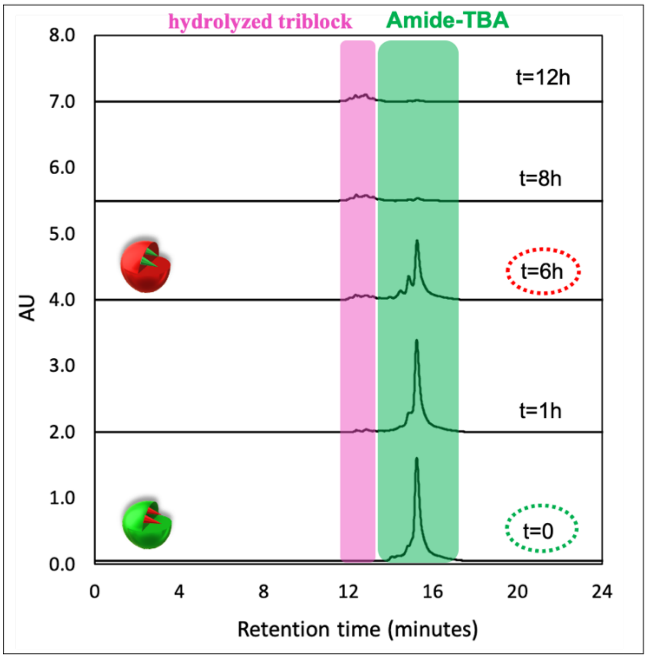

Figure S25: Overlay of HPLC chromatograms at 423 nm before and after the addition of PGA, followed by PLE (at t = 6 hours).

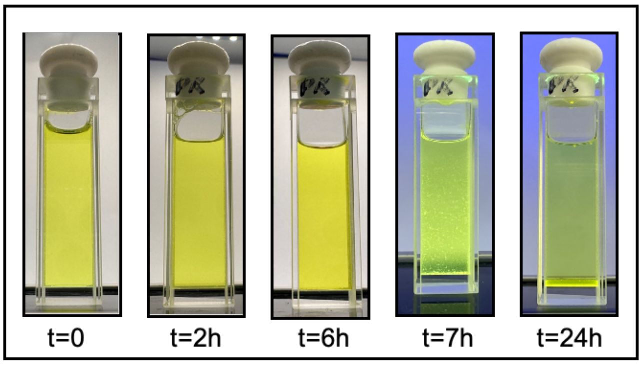

Figure S26: Photos of ester-DBA and amide-TBA formulation at different time points before and after the addition of PGA, followed by PLE (at t = 6 hours).

An additional experiment was also conducted in which we simply monitored the response of this formulation upon activation with PGA only but for an extended duration. The degradation was followed by HPLC at 297 nm, and each experiment was conducted thrice. The reported results are demonstrated in Figures 5D and 5E of the main text. The DLS measurement (Figure S28C) was done after 48 hours of incubation with PGA enzyme and didn't show any micellar presence. Pictures of the solution taken after 48 hours show the presence of a small amount of aggregated gel and that is why we decided to dilute the sample with acetonitrile (1:1 ACN:

PBS) and inject it into HPLC (Figure S28B). The HPLC analysis shows that we do have some amide-TBA that got aggregated into a gel during the enzymatic degradation process.

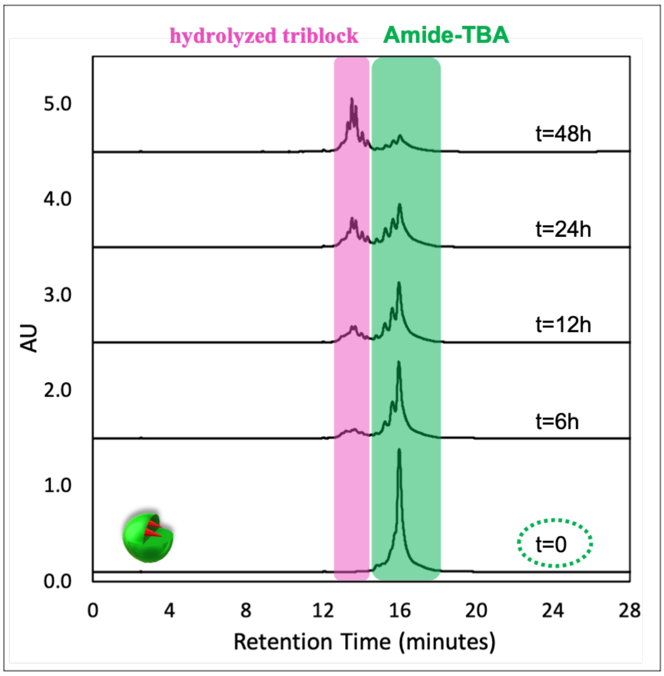

Figure S27: Overlay of HPLC chromatograms at 423 nm upon incubating solely with PGA for an extended duration (48 hours).

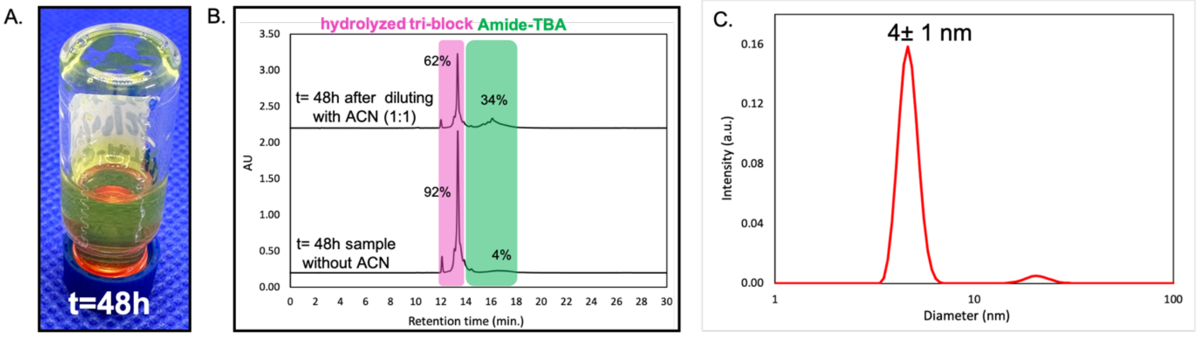

Figure S28: Ester-DBA and amide-TBA formulation: (A) A picture of the solution after 48 hours of incubation with PLE, (B) Overlay of HPLC chromatogram without adding acetonitrile and after adding acetonitrile (1:1 ACN: PBS) to the solution mixture, and (C) DLS measurement.

To monitor the thermodynamic stability of the mixed micelles obtained from ester-DBA and amide-TBA formulation, control experiments were done without adding an enzyme. The degradation was followed at 37 °C by monitoring the area under the peak of the parent amphiphile by HPLC at 297 nm. A picture of the solution taken at 24 hours indicates the stability of this formulation in the absence of an enzyme.

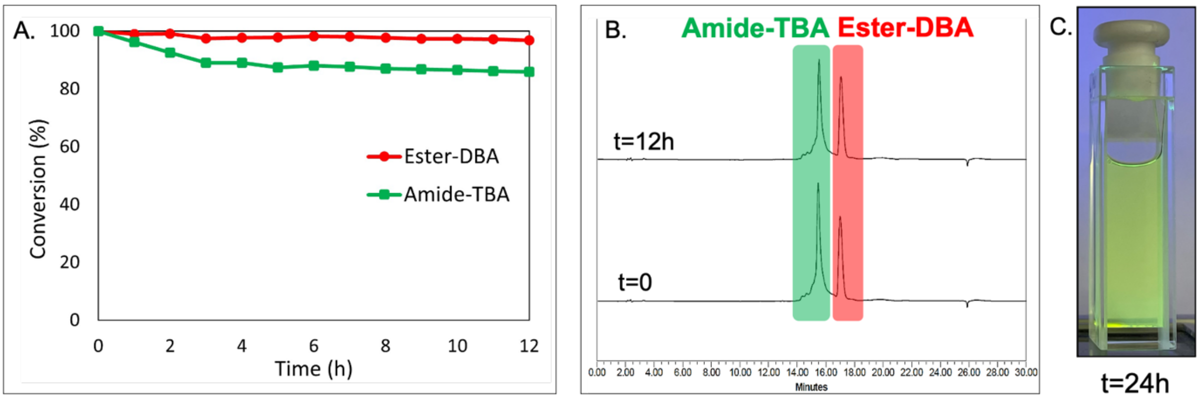

Figure S29: Ester-DBA and amide-TBA formulation in the absence of an enzyme (A) HPLC-based kinetics and (b) HPLC overlay, and (c) A picture of the solution.

5.4 Absorption measurements

The preparation of micellar solution for all three formulations was conducted using the same method, with enzyme concentrations identical to those used for the previous HPLC measurements. However, the absorption intensity was too high to monitor the degradation while maintaining absorbance linearity, given the concentration of the micellar solution. So, we decided to dilute 10 µL of the micellar solution with 240 µL of acetonitrile. Subsequently, we assessed the absorbance in a 96-well quartz plate and were able to bring it below 1 absorbance unit (AU), which represented the instrument's limit. Absorption spectra of the mixed labelled micelles were collected over a range of 300-600 nm.

To monitor the enzymatic degradation and triblock aggregation, we conducted absorbance every hour. We achieved this by taking out the 10 µL volume from the concentrated micellar solution and subsequently diluting it with acetonitrile. Following this, the diluted sample was then centrifuged to get rid of degraded enzymes, after which the absorbance was measured. We followed the same protocol every hour, spanning up to 10 hours, for all three formulations (Figures 1F, 1G, 2F, 2G, 4F, and 4G as shown in the main text).

6. <u>Characterization of the formed hydrogels and their further degradation</u>

6.1 Rheology measurements

Rheological measurements were performed using a controlled-stress rheometer (AR-G2, TA instruments, USA). An 8 mm diameter flat-plate geometry with a rough surface was used for the study. The viscous elastic region was determined by strain sweep from 0.01 to 100% strain at 1Hz frequency at 25°C, with a gap size of 0.9 mm.

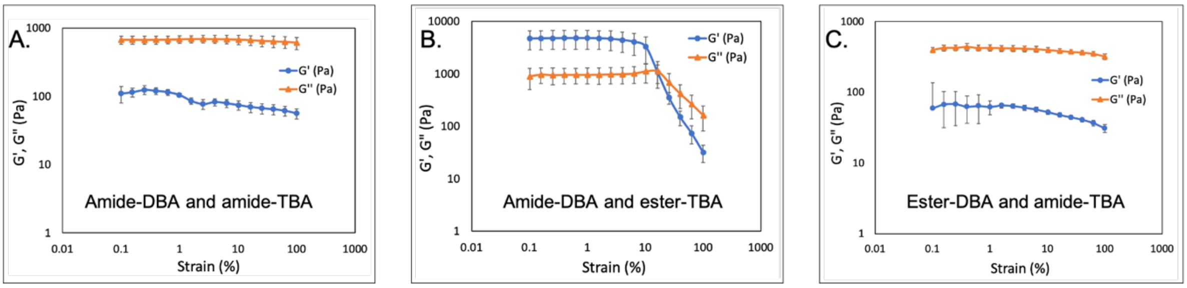

Figure S30: Amplitude sweep tests of the hydrogels obtained from all three formulations from 1:1 DBA: TBA weight ratio at a constant frequency of 1 Hz.

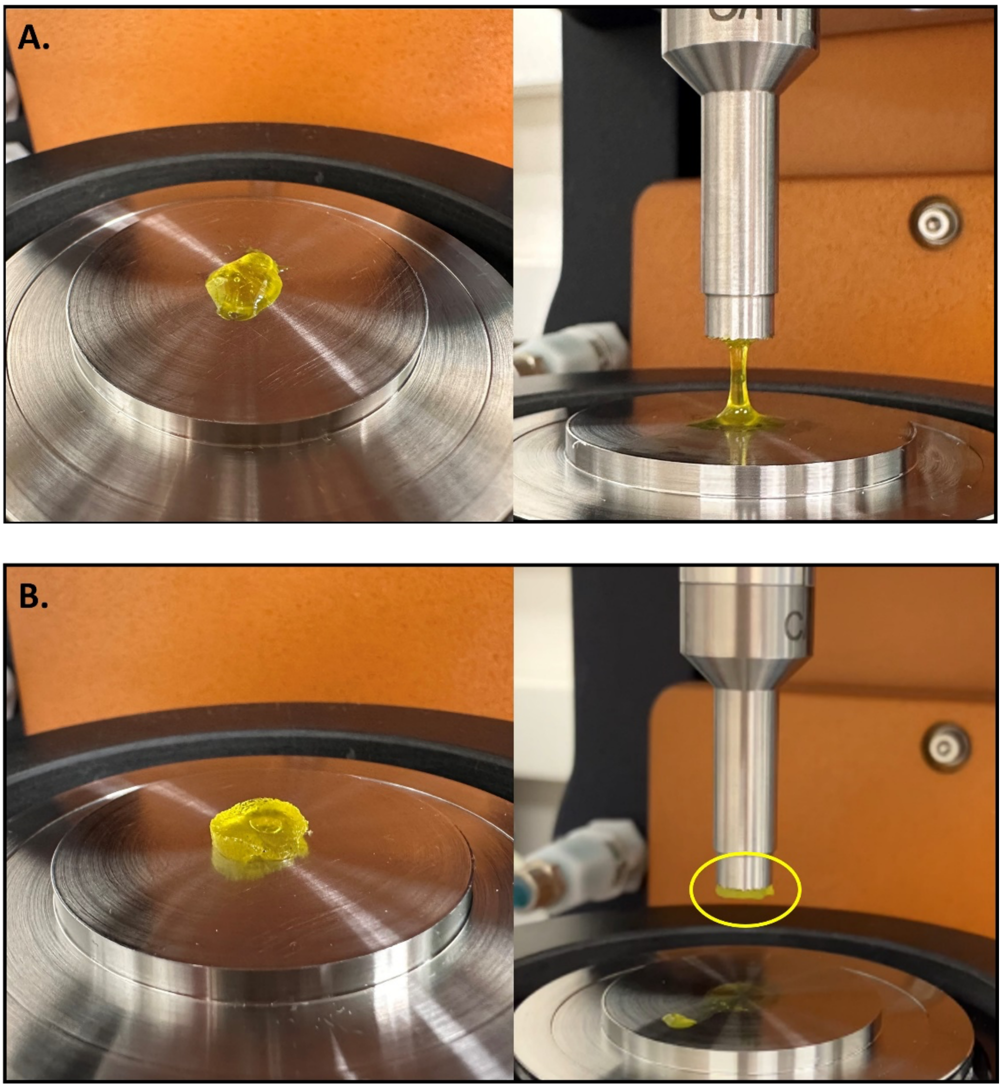

Figure S31: Pictures of the hydrogels formed from TBAs in water with a concentration of 5 mg/mL using thin-film hydration (A) Amide-gel (B) Ester-gel.

6.2 HRSEM measurements

All images were taken using a Zeiss Gemini 300 high-resolution scanning electron microscope in high vacuum, 15 kV.

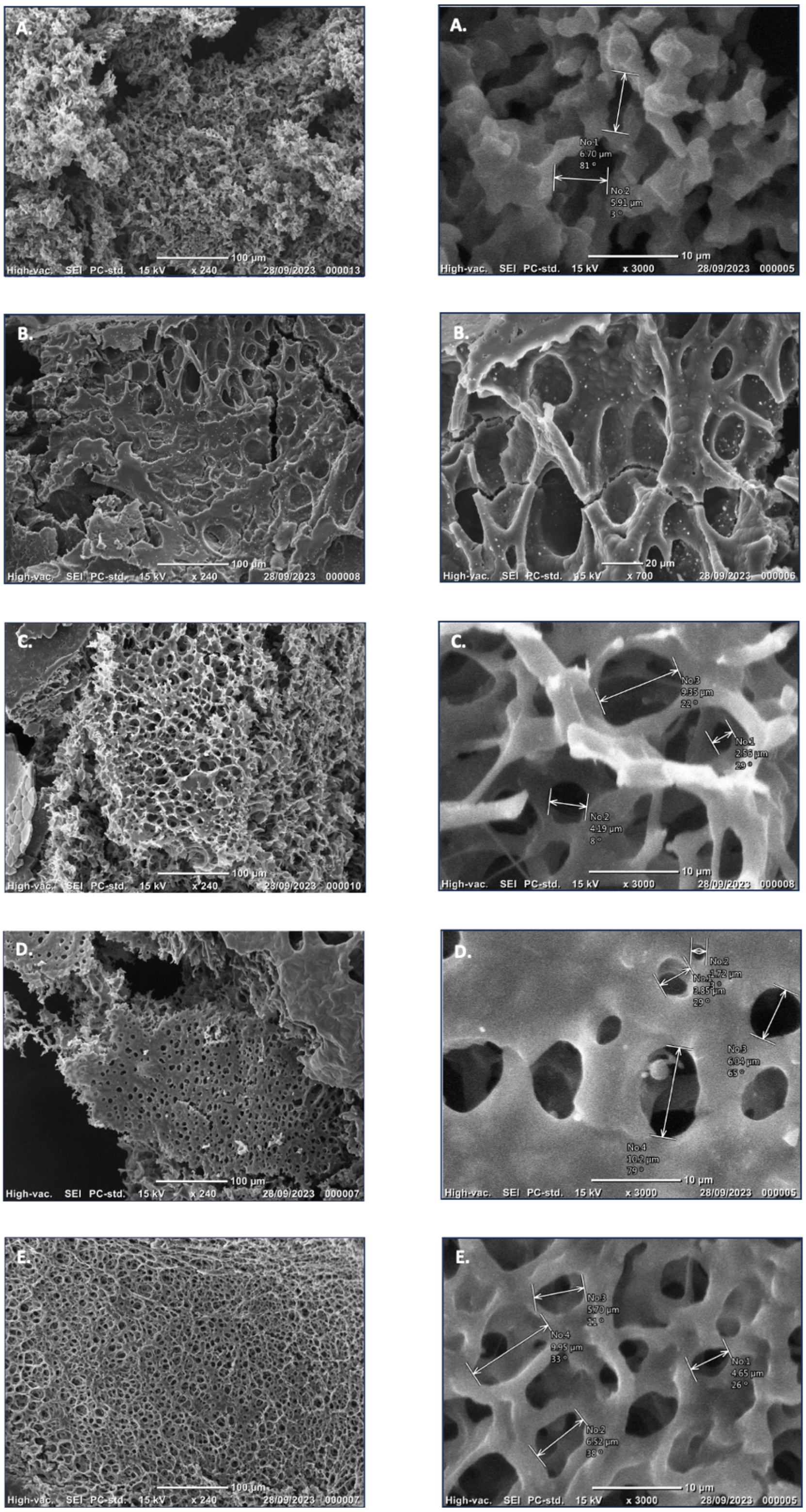

Figure S32: HRSEM images for hydrogels formed from TBAs at a concentration of 5 mg/mL. Left – scale bar: 100 µm and on right – zoomed-in. (A) Ester-TBA and (B) Amide-TBA. HRSEM images of gel aggregated from co-assembled micelles at a total polymer concentration of 10 mg/mL in 1:1 weight ratio. (C) Amide-DBA and amide-TBA, (D) Amide-DBA and ester-TBA, and (E) Ester-DBA and amide-TBA.

6.3 Small Angle X-Ray Scattering (SAXS)

Measurements were performed using an in-house X-ray scattering system, with a Genix3D (Xenocs) low divergence Cu K_α radiation source (wavelength of λ = 1.54 Å) and scatter-less slits setup.[5] Two-dimensional scattering data with a wave vector amplitude (q) range of 0.005−0.2 $Å^{-1}$ at sample-to-detector distance of ~1000 mm, were collected with an Eiger2 1M (Dectris) detector. The exact sample to detector position was calibrated using Silver behenate powder. The 2D diffraction data were radially integrated using data reduction software (SAXSi) developed in Beck's lab. Hydrogel samples were loaded inside 1.5 mm quartz capillaries (Hilgenberg). Acquisition time was typically 1800 s per frame. Peak position extracted from the 1D radially profiles.

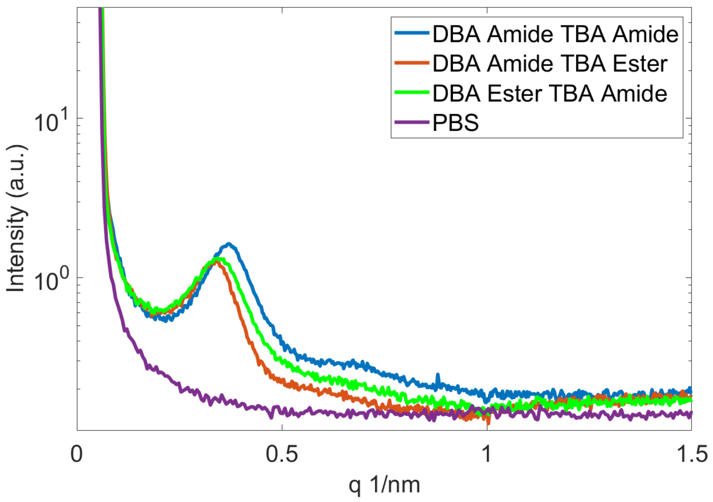

Figure S33: SAXS measurements of hydrogel samples. The scattering is dominated by a single structure-factor correlation peak. Peaks position for hydrogels formed from the following formulations: DBA-amide and TBA-amide (blue), DBA-amide and TBA-ester (green), and DBA-ester and TBA-amide (red) are q = 0.37 (1/nm), 0.35 (1/nm) and 0.34 (1/nm), respectively.

6.4 Hydrogel degradation

To study the stability of the hydrogel formed during the enzymatic degradation experiment (Section 5.1- 5.3), the solution above the hydrogel was removed, and the remaining hydrogel was washed three times with PBS. Two parallel experiments were conducted for all three different formulations. In the first experiment, 500 µL of 3.5 mg/mL BSA in PBS was added. In the second experiment, 500 µL of 3.5 mg/mL BSA, along with a higher concentration of PGA/PLE in PBS, was added. For the amide-based gel, PGA was added to yield a final concentration of 9.17 U/mL, while for the ester-based gel, PLE was added to achieve a final concentration of 35 U/mL. The results for hydrogel degradation formed from all three different formulations are presented in Figures 1I, 2I, and 4I of the main text.

6.5 Analysis of the composition of the formed hydrogels

The solution above the hydrogel formed during the enzymatic degradation experiment (Section 5.1-5.3), was removed and the remaining hydrogel was washed 3 times with PBS and then dissolved in acetonitrile. The HPLC analysis shows the presence of amide-TBA (Figure S34,1)

for the amide-DBA and amide-TBA formulation, while for the amide-DBA and ester-TBA formulation, we observed the presence of 6% hydrolyzed triblock polymers and 94% ester-TBA (Figure S35,1).

Once the gel in the vials containing both BSA and enzyme had completely degraded (Section 6.4), a 100 µL sample from the hydrolyzed polymer solution was mixed with 100 µL of acetonitrile to neutralize the BSA activity. Following this, the diluted sample was centrifuged to get rid of degraded enzymes/BSA and then injected into the HPLC for compositional analysis of the degraded gel. The same protocol was followed for the gel containing just BSA, aiming to determine if any degradation occurred under those conditions. The results of the HPLC analysis for both types of gels (amide-gel and ester-gel) under two parallel conditions - with BSA and BSA+ enzyme were presented in Figures S34 and S35.

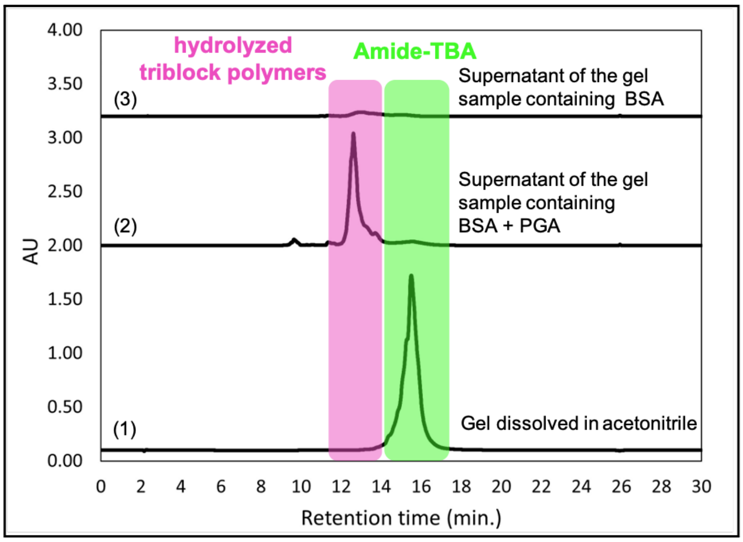

Figure S34: HPLC overlay: (1) after adding acetonitrile to the gel formed from amide-DBA and amide-TBA formulation, (2) hydrolyzed triblock polymers in the presence of BSA+PGA after 13 days, and (3) hydrolyzed triblock polymers in presence of BSA alone, after 13 days.

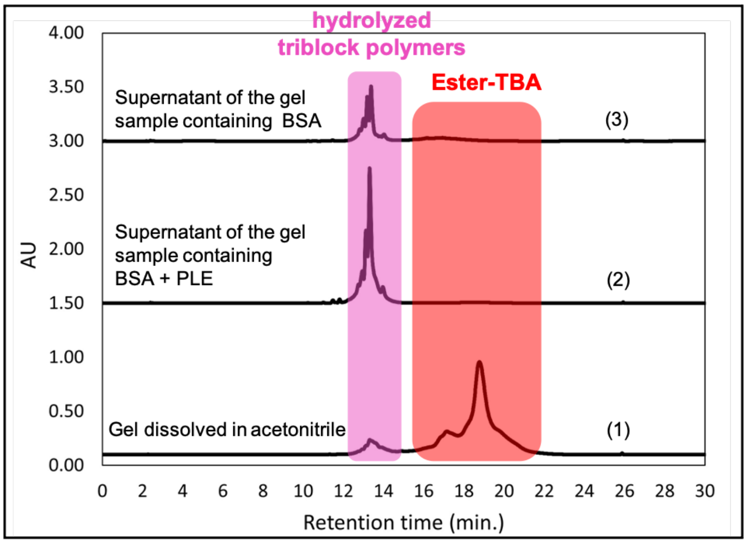

Figure S35: HPLC overlay: (1) after adding acetonitrile to the gel formed from amide-DBA and ester-TBA formulation, (2) hydrolyzed triblock polymers in the presence of BSA+PLE after 7 days, and (3) hydrolyzed triblock polymers in presence of BSA alone, after 7 days.

References:

(1) Slor, G.; Papo, N.; Hananel, U.; Amir, R. J. Tuning the Molecular Weight of Polymeric Amphiphiles as a Tool to Access Micelles with a Wide Range of Enzymatic Degradation Rates. *Chemical Communications* **2018**, *54* (50), 6875–6878. https://doi.org/10.1039/C8CC02415D.

(2) Harnoy, A. J.; Papo, N.; Slor, G.; Amir, R. J. Mixing End Groups in Thiol-Ene/Yne Reactions as a Simple Approach toward Multienzyme-Responsive Polymeric Amphiphiles. *Synlett* **2018**, *29* (19), 2582–2587. https://doi.org/10.1055/s-0037-1611340.

(3) Harnoy, A. J.; Buzhor, M.; Tirosh, E.; Shaharabani, R.; Beck, R.; Amir, R. J. Modular Synthetic Approach for Adjusting the Disassembly Rates of Enzyme-Responsive Polymeric Micelles. *Biomacromolecules* **2017**, *18* (4), 1218–1228. https://doi.org/10.1021/acs.biomac.6b01906.

(4) Rathee, P.; Edelstein-Pardo, N.; Netti, F.; Adler-Abramovich, L.; Sitt, A.; J. Amir, R. Architecture-Based Programming of Polymeric Micelles to Undergo Sequential Mesophase Transitions. *ACS Macro Lett* **2023**, *12* (6), 814–820. https://doi.org/10.1021/acsmacrolett.3c00153.

(5) Li, Y.; Beck, R.; Huang, T.; Choi, M. C.; Divinagracia, M. Scatterless Hybrid Metal-Single-Crystal Slit for Small-Angle X-Ray Scattering and High-Resolution X-Ray Diffraction. *J Appl Crystallogr* **2008**, *41* (6), 1134–1139. https://doi.org/10.1107/S0021889808031129.
